# Structural variation and DNA methylation shape the centromere-proximal meiotic crossover landscape in Arabidopsis

**DOI:** 10.1186/s13059-024-03163-4

**Published:** 2024-01-22

**Authors:** Joiselle B. Fernandes, Matthew Naish, Qichao Lian, Robin Burns, Andrew J. Tock, Fernando A. Rabanal, Piotr Wlodzimierz, Anette Habring, Robert E. Nicholas, Detlef Weigel, Raphael Mercier, Ian R. Henderson

**Affiliations:** 1 Department of Plant Sciences, University of Cambridge, Cambridge, CB2 3EA UK; 2 Department of Chromosome Biology, Max Planck Institute for Plant Breeding Research, D-50829 Cologne, Germany; 3 Department of Molecular Biology, Max Planck Institute for Biology, Tübingen, D-72076 Tübingen, Germany; 4 University of Tübingen, Institute for Bioinformatics and Medical Informatics, D-72076 Tübingen, Germany

**Keywords:** Centromeres, Meiosis, Crossover, Recombination, DNA methylation, Arabidopsis

## Abstract

**Background:**

Centromeres load kinetochore complexes onto chromosomes, which mediate spindle attachment and allow segregation during cell division. Although centromeres perform a conserved cellular function, their underlying DNA sequences are highly divergent within and between species. Despite variability in DNA sequence, centromeres are also universally suppressed for meiotic crossover recombination, across eukaryotes. However, the genetic and epigenetic factors responsible for suppression of centromeric crossovers remain to be completely defined.

**Results:**

To explore the centromere-proximal meiotic recombination landscape, we map 14,397 crossovers against fully assembled *Arabidopsis thaliana* (*A. thaliana*) genomes. *A. thaliana* centromeres comprise megabase satellite repeat arrays that load nucleosomes containing the CENH3 histone variant. Each chromosome contains a structurally polymorphic region of ~3–4 megabases, which lack crossovers and include the satellite arrays. This polymorphic region is flanked by ~1–2 megabase low-recombination zones. These recombination-suppressed regions are enriched for Gypsy/Ty3 retrotransposons, and additionally contain expressed genes with high genetic diversity that initiate meiotic recombination, yet do not crossover. We map crossovers at high-resolution in proximity to *CEN3*, which resolves punctate centromere-proximal hotspots that overlap gene islands embedded in heterochromatin. Centromeres are densely DNA methylated and the recombination landscape is remodelled in DNA methylation mutants. We observe that the centromeric low-recombining zones decrease and increase crossovers in CG (*met1*) and non-CG (*cmt3*) mutants, respectively, whereas the core non-recombining zones remain suppressed.

**Conclusion:**

Our work relates the genetic and epigenetic organization of *A. thaliana* centromeres and flanking pericentromeric heterochromatin to the zones of crossover suppression that surround the CENH3-occupied satellite repeat arrays.

**Supplementary Information:**

The online version contains supplementary material available at 10.1186/s13059-024-03163-4.

## Background

Meiosis is a specialized eukaryotic cell division where a single round of DNA replication is coupled to two rounds of chromosome segregation, to produce haploid gametes [1, 2]. During the first meiotic division, homologous chromosomes physically pair and undergo recombination that can result in reciprocal genetic exchange, termed crossover [1–3]. Meiotic recombination and independent chromosome segregation increase genetic diversity by reshuffling parental genomes into the gametes [1, 2]. Meiotic recombination is initiated via DNA double-strand breaks (DSBs) catalyzed by SPO11 complexes [2, 4, 5]. SPO11-dependent DSBs are resected to form single-stranded DNA, which can then mediate strand invasion of a sister or homologous chromosome and be repaired as a reciprocal crossover, or non-reciprocal non-crossover [2, 4, 5]. Two major pathways, termed Class I and Class II, are required for crossover formation in plants [2]. Class I crossovers are the numerical majority and also show the phenomenon of interference, where double crossovers are more widely spaced than expected at random, whereas Class II events do not show interference [1, 2]. Meiotic recombination occurs during prophase-I, when replicated homologs are physically associated via a chromosome axis and the synaptonemal complex, which provide the physical context for recombination [1–3].

Meiotic recombination frequency is highly variable within eukaryotic genomes, and kilobase-scale hotspots of both DSBs and crossovers exist in plants, animals, and fungi, whose locations are defined by a combination of DNA sequence and epigenetic information [6, 7]. Conversely, other genomic regions are strongly crossover-suppressed, including the centromeres, repetitive heterochromatin, mating-type loci, and sex chromosomes [8–11]. It has been proposed that suppression of crossovers within and around centromeres is beneficial, as proximal exchanges are associated with aneuploidy in fungi and animals, including trisomy in humans [12–15]. In addition, crossovers may lead to non-allelic exchanges in repeat regions, with the potential to cause deleterious structural change [11].

The centromeres function to assemble the kinetochore complex, which mediates chromosome attachment to spindle microtubules, during mitotic and meiotic cell divisions [16]. Centromere DNA sequences are loaded with nucleosomes containing the CENH3/CENP-A histone variant, which assemble the kinetochore [17, 18]. Despite a conserved role in CENH3/CENP-A loading, centromere DNA sequences are highly divergent within and between species [19–21], ranging from a ~120 base pair (bp) sequence in budding yeast, to megabase satellite repeat arrays in plants and animals [8, 21–23]. Although eukaryotic centromeres are composed of diverse DNA sequences, all known centromeres show meiotic crossover suppression that spreads into flanking regions, over distances of kilobases to megabases [8, 10, 12, 22]. However, the genetic and epigenetic features that regulate centromere-proximal recombination are incompletely understood.

Long-read DNA sequencing technologies, including PacBio HiFi and Oxford Nanopore, have allowed complete assembly of complex repeat regions [22–24]. For example, long-read DNA sequencing led to the assembly of the *Arabidopsis thaliana* centromeres, which comprise megabase arrays of a 178-bp tandem repeat (*CEN178*) that are the site of CENH3 loading [21, 22, 24]. Plant and animal centromeres are often densely cytosine methylated, although the specific pattern varies between species [22, 23, 25]. For example, the CENP-A occupied regions of human α-satellite centromere arrays show CG context DNA hypomethylation [26]. In contrast, the Arabidopsis CENH3-enriched regions are densely CG methylated, but hypomethylated in the CHG context [22]. In Arabidopsis, CG and non-CG context DNA methylation are maintained by distinct methyltransferase enzymes; MET1 for CG, and CMT2, CMT3, and DRM2 for non-CG [27]. The Arabidopsis pericentromeric regions are dominated by transposable elements and are also enriched for heterochromatic chromatin marks including H3K9me2, H3K27me1, H2A.W6, H2A.W7, and the meiotic cohesin REC8 [28–30]. Using complete maps of the Arabidopsis genome, we sought to investigate how genetic and epigenetic information shape the crossover landscape in proximity to the centromeres.

We mapped 14,397 crossovers genome-wide, against complete assemblies of the Arabidopsis Col and Ler accessions, and precisely identified zones of centromere-proximal suppressed recombination. The crossover-suppressed zones contain structurally variable satellite repeat arrays that are densely DNA methylated and load CENH3 nucleosomes, which we propose exert a joint suppressive effect on the recombination landscape. Low-recombining zones flank the centromeres and contain expressed genes that show elevated genetic diversity, with a range of housekeeping and environment-response functions. These centromere-proximal genes show evidence for meiotic recombination initiation, but not crossovers, indicating that repair steps downstream of DSB formation are inhibited. Using a fluorescence-based selection strategy, we fine-mapped 913 crossovers in proximity to *CEN3* and observed punctate recombination hotspots that overlap gene islands embedded in pericentromeric heterochromatin. We additionally mapped 962 and 1033 *CEN3*-proximal crossovers in mutants defective in maintenance of CG (*met1*), or CHG (*cmt3*) sequence context DNA methylation. Centromere-proximal crossovers decreased and increased in *met1* and *cmt3*, respectively, and fine-scale remodelling of the recombination landscape was observed, although the satellite arrays remained crossover-suppressed in both cases. Our maps provide functional insight into the genetic and epigenetic factors that shape recombination in proximity to the Arabidopsis centromeres.

## Results

### The landscape of centromere-proximal crossover frequency in Arabidopsis

The Arabidopsis Col-0 (hereafter Col) and Ler-0 (hereafter Ler) accessions are of Eurasian origin and show ~0.5% difference between shared DNA sequences in the chromosome arms [31]. Short-read sequencing of F_2_ or backcross progeny from Col/Ler F_1_ parents has been used to map meiotic crossovers [9, 32–36]. As the Col and Ler centromere sequences have been fully assembled using long-read DNA sequencing [21, 22, 24], we sought to utilize these genome maps with the available crossover data to examine centromere-proximal recombination. For analysis, we combined 1009 crossovers mapped from a Col/Ler female BC_1_ population, 978 crossovers from a male BC_1_ population, and 12,410 from an F_2_ population, giving 14,397 crossovers in total and representing 3613 meioses [9, 33]. For crossover mapping, we used a refined set of single-nucleotide polymorphisms (SNPs) that had been filtered for quality, and Mendelian segregation ratios in recombinant populations (Additional file 1: Fig. S1A). Against the Col-CEN assembly, the average filtered Col/Ler SNP density was 2.56 per kb, and crossovers were resolved to a median width of 3992 bp (Additional file 1: Fig. S1B). Due to structural polymorphism and the challenges of sequence alignment within the centromeres, few SNPs were identified within the *CEN178* array regions (Additional file 1: Fig. S1C). Following short-read alignment from each individual library, the refined Col/Ler SNPs were genotyped and a sliding window approach used to map crossover intervals (Additional file 1: Fig. S1C) [33–35].

**Fig. 1:**
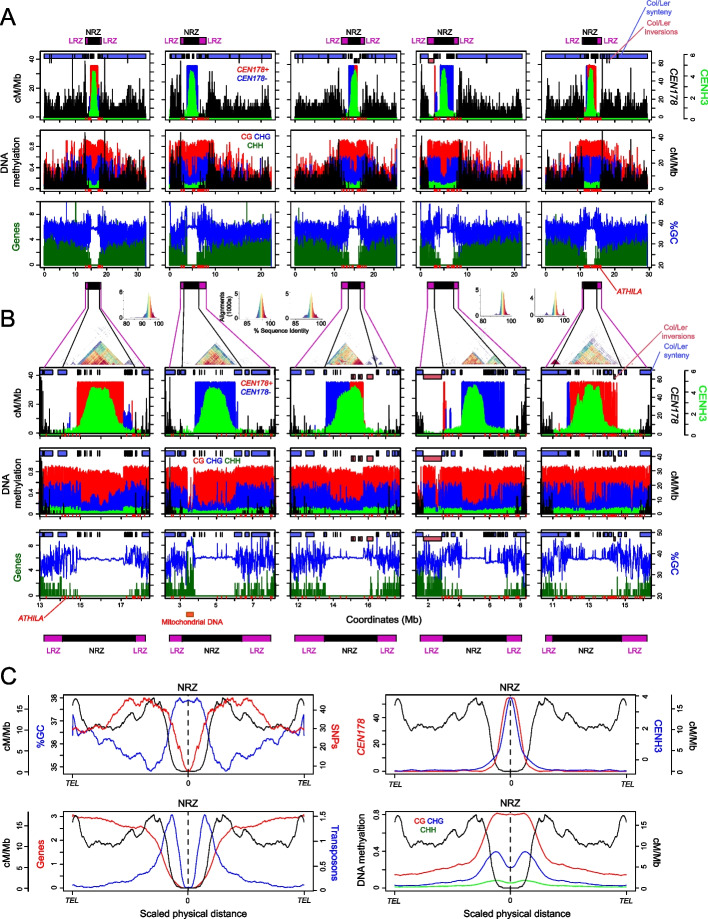
Zones of centromere-proximal crossover suppression in Arabidopsis. **A** In the first row, Col/Ler crossover frequency (cM/Mb) is plotted against the Col-CEN assembly in 10-kb windows (black). *CEN178* satellite density (red=forward & blue=reverse strand) and CENH3 ChIP-seq enrichment (green, log_2_[ChIP/input]) are plotted in the same windows. Locations of the non-recombining zones (NRZ, black), and low-recombining zones (LRZ, purple), are indicated above. Shaded blocks at the top of the plots indicate regions of Col/Ler synteny (blue) and inversions (pink) mapped by SyRI [37]. In the next row, the proportion of DNA methylation mapped from ONT reads is plotted in 10-kb windows for CG (red), CHG (blue), and CHH (green) sequence contexts. Beneath, the density of genes (green) is plotted alongside %GC content (blue) per 10 kb. *ATHILA* retrotransposon insertions are indicated as red *x*-axis ticks. **B** As for **A**, but showing a zoom of the NRZ and LRZ regions. Plot annotations are the same, apart from a StainedGlass sequence identity heat map is positioned over the plots [38], and NRZ-LRZ positions are shown beneath. Histograms showing the frequency of alignments, and color correspondences, with different % sequence identity are shown above the StainedGlass heat maps. **C** Quantification of genomic features plotted along the ten chromosome arms that were proportionally scaled between telomeres (*TEL*) and NRZ midpoints. Data analyzed were gene, transposon and *CEN178* density per 10 kb, CENH3 log_2_(ChIP/input), %GC base composition, DNA methylation, and crossovers (cM/Mb). Information on chromatin datasets analyzed is available in Additional file 6: Table S2

To define zones of centromere-proximal recombination suppression, we tallied crossovers in 10-kb windows and defined (i) the Non-Recombining Zones (NRZs) as contiguous centromeric regions with an absence of crossovers and (ii) the Low-Recombining Zones (LRZs) as the flanking windows within 1 cM of each NRZ boundary (Fig. 1, Additional file 1: Fig. S1C and Additional file 2: Table S1). One-centiMorgan windows were selected to define the LRZs based on genetic map length, rather than physical distance, on the different chromosomes. LRZ boundaries are robust to sampling, whereas NRZ boundaries are sensitive to rare crossover events. In total, the Col-CEN NRZs span 17.8 Mb and are flanked by 10.8 Mb of LRZs (Fig. 1, Additional file 1: Fig. S1C and Additional file 2: Table S1). The mean density of SNPs in the LRZs (3.85 SNPs/kb) was higher than in the chromosome arms (2.80 SNPs/kb), meaning we are not limited by SNPs for detection of crossovers in the LRZs. SNP density is lower in the NRZs (0.75 SNPs/kb), which will reduce the precision of mapping individual crossovers, but not the ability to detect them, as flanking markers are sufficient to detect intervening crossovers. The LRZs comprise 10 cM in total with a recombination rate of 0.93 cM/Mb, compared to 3.80 cM/Mb in the chromosome arms. The majority of the NRZs are composed of the *CEN178* satellite arrays (13.2 Mb, or 74%), and additionally contain mitochondrial genome insertions, *5S*rDNA, telomere, and *CEN159* repeat arrays (Fig. 1, Additional file 1: Fig. S1 and Additional file 2: Table S1) [22]. Within each Col-CEN *CEN178* satellite array, 1.4–1.6-Mb regions show CENH3 log_2_(ChIP/Input) enrichment scores >2, indicating the kinetochore locations (Fig. 1 and Additional file 2: Table S1). We conclude that centromeric crossover inhibition spreads significantly beyond the boundaries of the CENH3-occupied regions (mean=1.76 Mb), and the *CEN178* satellite arrays (mean=2.54 Mb), with the joint LRZ-NRZ spanning on average 5.72 Mb per chromosome (Fig. 1 and Additional file 2: Table S1).

To compare crossover distributions in the Ler genome, we used the 14,397 crossover locations mapped against the Col-CEN genome and extracted 2 kb of sequence centered on each crossover midpoint. These sequences were used to perform alignments against the Ler-HiFi assembly using LASTZ [39]. Of the 14,397 crossovers, only four could not be aligned using this method. For each crossover, we selected the alignment with highest coverage, and removed crossovers that aligned to multiple loci with equally high coverage or identity values. Using the 13,958 high-confidence crossovers alignments, we calculated NRZ and LRZ coordinates, as previously. The Ler NRZs are 16.3 Mb, compared to 17.8 Mb in Col, whereas the Ler LRZs are 13.9 Mb, compared to 10.8 Mb in Col (Additional file 3: Fig. S2 and Additional file 2: Table S1), indicating that the extent of centromere-proximal recombination suppression is similar in both genomes.

The Col-CEN LRZs and NRZs are densely DNA methylated in CG, CHG, and CHH sequence contexts, although the CENH3-enriched regions within the NRZs show relative depletion of CHG context methylation (Fig. 1A–C). CHG depletion in the centromeres is proposed to be a consequence of CENH3 being unable to sustain H3K9me2 histone methylation, which is required to maintain CHG context DNA methylation in Arabidopsis [22, 27]. The Col-CEN LRZs are strongly enriched for heterochromatic histone modifications H3K9me2, H3K27me1, H2A.W6, and H2A.W7, although similar to CHG DNA methylation, these marks are relatively depleted within NRZ-CENH3 regions (Additional file 4: Fig. S3) [28, 40]. Interestingly, H2A.W7 showed a stronger depletion in the CENH3-enriched regions compared to H2A.W6 (Additional file 4: Fig. S3). Both Col-CEN LRZs and NRZs have significantly higher GC base content (37.6 and 38.2%), compared to the chromosome arms (35.8%) (Wilcox test *P*=0.0079) (Fig. 1). ChIP-seq enrichment of REC8-cohesin, and the HORMA domain protein ASY1, which are components of the meiotic chromosome axes, are strongly enriched in the Col-CEN LRZs and NRZs, and to a lesser extent within the NRZ-CENH3 regions (Additional file 5: Fig. S4) [28, 41]. We detected peaks of SPO11-1-oligos, a marker of meiotic DNA double-strand breaks, within the LRZs, which correlated with crossovers in a subset of cases (Additional file 5:Fig. S4) [42]. SPO11-1-oligo peaks observed in the absence of crossovers may reflect initiation of meiotic DSBs, but repression of downstream crossover repair by centromeric chromatin states, or structural polymorphism. Information on all chromatin datasets analyzed is available in Additional file 6: Table S2.

To explore patterns of centromeric structural polymorphism, we used Synteny and Rearrangement Identifier (SyRI) to identify syntenic regions between the Col and Ler genomes, in addition to rearrangements (Fig. 1A,B) [37]. The LRZs-NRZs show disrupted synteny between Col and Ler, consistent with structural polymorphism contributing to centromere-proximal crossover suppression (Fig. 1A,B). This includes inversion of the chromosome 3 *CEN178* array and flanking sequences, and a large pericentromeric “knob” inversion adjacent to *CEN4* (Fig. 1A,B) [22, 43]. We compared the structure of the satellite arrays between Col and Ler, for each chromosome, and observed significant structural polymorphism, despite them being composed of the same *CEN178* repeats (Fig. 1 and Additional file 7: Fig. S5). For example, both *CEN1*and *CEN2* are larger (2.67 and 2.91 vs 1.91 and 1.51 Mb) and more repetitive in Col (Additional file 7: Fig. S5). Col and Ler *CEN3* show array inversions, but the Ler centromere is larger and consists of discontinuous islands of repeats on its left boundary (Additional file 7: Fig. S5). In both accessions, *CEN4* consists of two adjacent, yet distinct, *CEN178* arrays, with the left array being CENH3-occupied and more divergent in sequence between the accessions (Additional file 7: Fig. S5). In contrast*, CEN5* is larger and more repetitive in Ler (3.37 vs 2.66 Mb) (Additional file 7: Fig. S5). The extensive *CEN178* repeat array polymorphisms, including inversions, between the Col and Ler genomes could directly contribute to NRZ crossover suppression, in addition to the effects of epigenetic information.

### Gene and transposon content of the centromeric recombination-suppressed zones

**Fig. 2:**
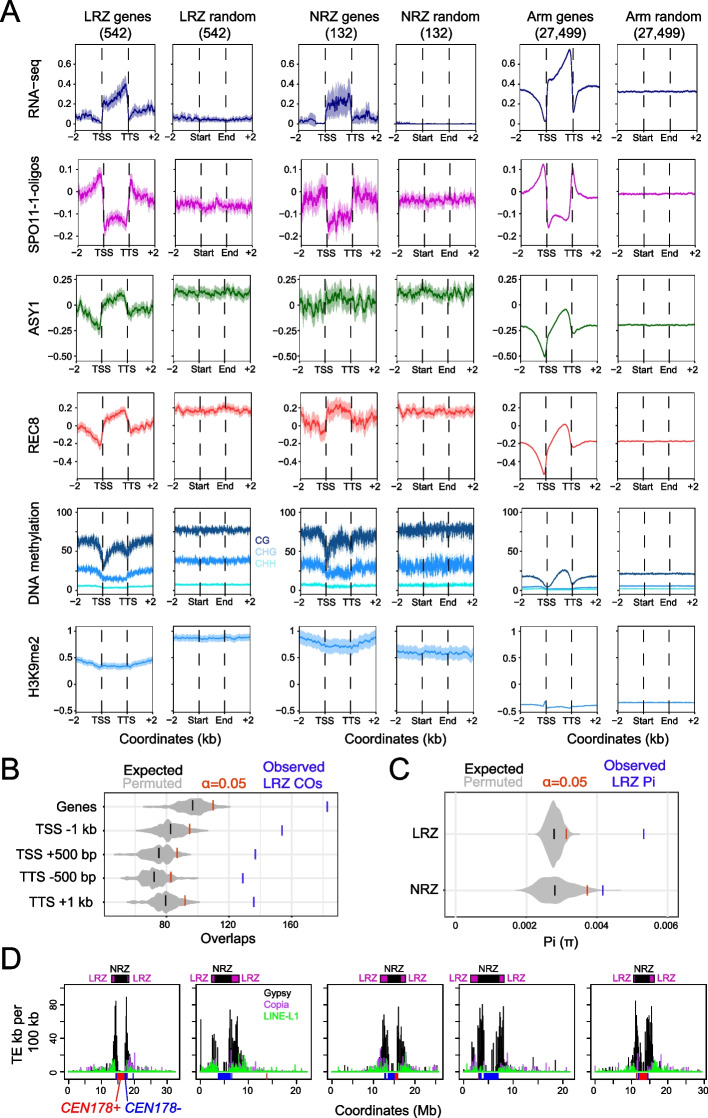
Gene and transposon content of the Arabidopsis LRZs and NRZs. **A** Meta-profiles of leaf RNA-seq (blue, Log_2_[RNA-seq (TPM)], SPO11-1-oligos (pink, Log_2_[SPO11-1-oligos/genomic DNA]), ASY1 (green, Log_2_[ChIP-seq/input]), REC8 (red, Log_2_[ChIP-seq/input]), DNA methylation (%, CG=dark blue, CHG=blue, CHH=light blue), and H3K9me2 (blue, Log_2_[ChIP-seq/input]), across genes located in the chromosome arms (*n*=27,499), the LRZs (*n*=542), and the NRZs (*n*=132) and in 2-kb flanking regions. For each gene set, the same number of random windows of the same widths was compared within the same regions. Plot ribbons denote 95% confidence intervals for windowed values. Information on chromatin datasets analyzed is available in Additional file 6: Table S2. **B** Observed number of LRZ crossovers overlapping the listed gene features are shown (blue), compared to 10,000 sets of randomly positioned loci of the same number and width distribution as the LRZ crossovers. The *α*=0.05 significance thresholds are indicated (red), and the means of the permuted sets of loci (black) (*P*-values from all comparisons were <0.0001). **C** The observed median Pi (*π*) value for genes located in the LRZs (*n*=336) and NRZs (*n*=58) (blue), compared to 1000 sets of randomly chosen genes in the chromosome arms (gray). *α*=0.05 significance thresholds are indicated (red), and the medians of the permuted loci sets (black). *P*-values for both comparisons were <0.0001. Pi was calculated using 1001 Genomes Project SNPs [46]. **D** Plots of retrotransposon density per 100 kb along the Col-CEN assembly, showing Gypsy (black), Copia (purple), and LINE (green) superfamilies. The locations of the LRZs (purple) and NRZs (black) are indicated above the plots, and the *CEN178* satellite arrays (red, blue) are indicated along the *x*-axis

The Col-CEN LRZs and NRZs contain 542 and 132 genes, respectively, excluding the mitochondrial genes (*n*=106) located adjacent to *CEN2* [44]. The LRZs and NRZs have lower gene density (50 and 7 genes/Mb, respectively), compared to the chromosome arms (268 genes/Mb). Despite the Col-CEN NRZs and LRZs being heterochromatic, 42 and 48% of the contained genes showed evidence of expression from RNA-seq data, respectively (Fig. 2A and Additional file 8: Table S3) [45]. The NRZ and LRZ genes included those with housekeeping and environment-response annotation, genes with genetically defined roles (e.g., *ARP6*, *CLASSY1*, *MBD12*, and *OSCA1*), and with putative roles in immunity (e.g., defensins, *TIR-NBS14*, and WRKY transcription factors) (Additional file 8: Table S3). We also observed eight gene clusters, comprised of at least four orthologs, that encode calcium-dependent kinases, ankyrin-repeats, nucleotide transporters, receptor-kinases, ubiquitin, F-box proteins, sulfoxide reductases, and lipid-transfer proteins (Additional file 8: Table S3). Hence, the centromere-proximal recombination-suppressed regions contain genes with a diversity of predicted functions.

The Col-CEN LRZ and NRZ genes showed comparable accumulation of SPO11-1-oligos in their promoters and terminators, compared to chromosome arm genes (Fig. 2A) [42]. Chromosome arm gene bodies are enriched for REC8-cohesin and ASY1, which was also observed for LRZ and NRZ genes, although levels were higher in the LRZ/NRZ genes (Fig. 2A) [28, 41]. DNA methylation levels were higher in the NRZ and LRZ genes compared to the chromosome arms, yet they retained a typical profile of relative DNA hypomethylation in promoters and terminators and CG context methylation within their open reading frames (Fig. 2A). One striking difference between the Col-CEN NRZ/LRZ genes and those in the chromosome arms is greater enrichment of the heterochromatic H3K9me2 histone modification (Fig. 2A) [28]. As H3K9me2 has been associated with crossover suppression in Arabidopsis [47, 48], we propose this contributes to inhibition of crossover repair, despite significant SPO11-1-oligos forming in the NRZ and LRZ gene promoters and terminators (Fig. 2A).

Although Col-CEN LRZ crossovers are relatively suppressed compared to the chromosome arms, we investigated whether they locally overlapped genes. We compared observed overlaps between crossovers and LRZ genes, with overlaps of randomly positioned loci. For statistical comparison, 10,000 permuted randomly positioned sets within the LRZs of the same number and widths as the crossovers were used (Fig. 2B). We observed that LRZ crossovers significantly overlapped with genes and their upstream and downstream regions (all tests *P*=9.99⨉10^−5^) (Fig. 2B). We also analyzed transposon content within the NRZs and LRZs and observed strong enrichment of Gypsy/Ty3 LTR class retrotransposons (151.0 and 73.2 per Mb, respectively), compared to the chromosome arms (12.0 per Mb) (Fig. 2D). This implies that Gypsy/Ty3 retrotransposons may preferentially integrate into the LRZs and NRZs, or their insertions have been selected against in the chromosome arms. We repeated permutation tests for crossover overlap with LRZ transposons and observed significant overlap with Helitron transposons, and significant non-overlap with Gypsy/Ty3 retrotransposons (both *P*≤9.00⨉10^−5^), consistent with previous positive and negative associations of these families with meiotic DSBs, respectively [42].

We examined NRZ and LRZ gene sequence diversity compared to the chromosome arms. We calculated pairwise diversity (Pi, *π*) for genes, using SNPs from 1135 Arabidopsis accessions [46]. The SNPs were masked for repeated sequences, and we required that at least half of the gene had sequencing coverage across the 1135 accessions to be included. We also excluded genes that overlapped Col/Ler inversions, and the mitochondrial genome insertions on chromosome 2. After filtering, we retained 336 LRZ and 58 NRZ genes for analysis, calculated median *π*, and compared to 1000 permutations of the same numbers of genes from the chromosome arms (Fig. 2C). The observed median value of *π* for genes located in the LRZs and NRZs were significantly higher than the permuted sets from the chromosome arms (both *P*≤0.0001) (Fig. 2C). However, further work will be required to ascertain the relative contributions of recombination, demography, mutation rate, and selection to elevated LRZ and NRZ gene diversity.

### Fine-mapping of meiotic crossovers in proximity to *CENTROMERE3*

**Fig. 3:**
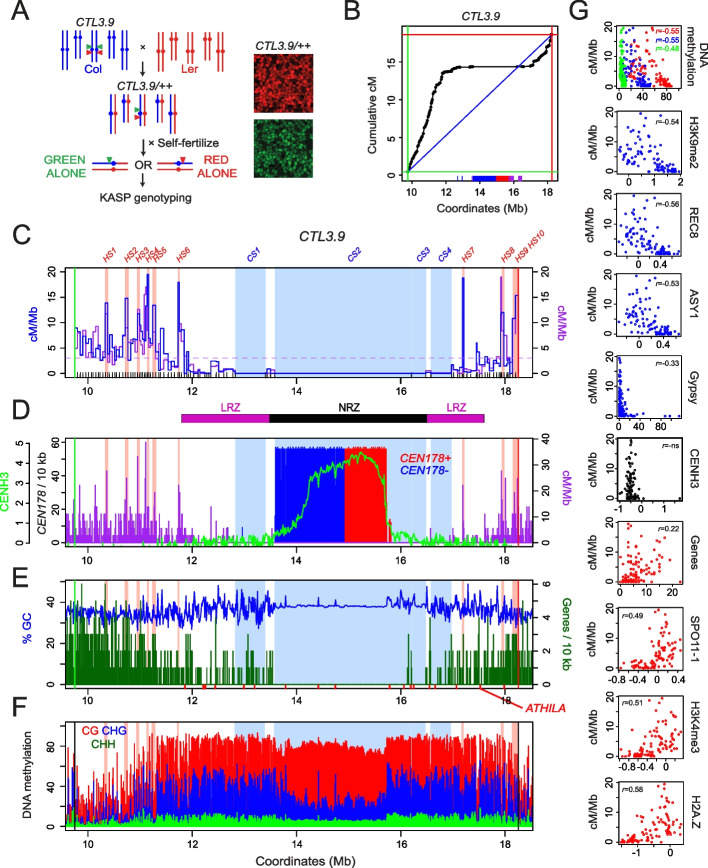
The fine-scale recombination landscape around Arabidopsis centromere 3. **A** Genetic strategy to recover crossovers within the *CTL3.9* FTL interval. FTL T-DNAs encoding red and green fluorescent proteins are indicated by triangles. The parental chromosomes are from the Col (blue) and Ler (red) accessions. Fluorescent micrographs of *CTL3.9/++* segregating seed are shown to the right. **B** Cumulative genetic map (centiMorgans, cM) relative to *CTL3.9* genomic coordinates plotted against the Col-CEN assembly derived from KASP genotyping of selected recombinant seed. The blue diagonal line shows a linear relationship, with the red and green lines showing the *CTL3.9* T-DNAs. The position of *CEN178* satellite repeats are shown as red (forward) and blue (reverse) ticks on the *x*-axis, in addition to *5S* rDNA (purple). **C** Crossover frequency (centiMorgan per megabase, cM/Mb, blue) plotted within *CTL3.9* derived from KASP genotyping and compared to measurements from mapping-by-sequencing (purple) for the same intervals. FTL T-DNAs are indicated by red and green vertical lines. Col/Ler KASP marker positions are indicated as *x*-axis ticks. The horizontal dotted line shows the genome average cM/Mb. Map intervals with significantly higher (*HOTSPOT, HS*) or lower (*COLDSPOT*, *CS*) crossover counts are shaded pink and blue, respectively. The black and purple bars beneath show the NRZ and LRZs. **D** As for **C**, but the frequency of *CEN178* satellite repeats on forward (red) and reverse (blue) strands per 10 kb is shown, overlaid with CENH3 log_2_(ChIP/input) enrichment (green) and GBS cM/Mb (purple). **E** As for **C**, but showing gene density per 10 kb (green), overlaid with %GC content (blue). *ATHILA* retrotransposon positions are indicated by *x*-axis ticks (red). **F** As for **C**, but showing the proportion of DNA methylation in 10-kb windows for CG (red), CHG (blue), and CHH (green) contexts. **G** cM/Mb values for *CTL3.9* map intervals are presented as scatter plots compared against DNA methylation, H3K9me2, REC8, ASY1 ChIP-seq, Gypsy/Ty3 transposons, CENH3 ChIP-seq, genes, SPO11-1-oligonucleotides, H3K4me3, and H2A.Z. Spearman’s correlation coefficient is printed inset, where significant. Information on chromatin datasets analyzed is available in Additional file 6: Table S2

As centromere-proximal crossovers are rare, we designed a fluorescence-based selection strategy to enrich for recombination events and perform fine-mapping (Fig. 3A). In Arabidopsis, linked hemizygous T-DNAs (fluorescence-tagged lines, FTLs) expressing different colors of fluorescent protein in the pollen or seed can be used to quantify and map intervening crossovers (Fig. 3A) [49, 50]. We selected the *CTL3.9* FTL, which was generated in the Col accession, to investigate crossover frequency in proximity to *CEN3* (Fig. 3A–G) [50]. Against the Col-CEN assembly, the *CTL3.9* T-DNAs define an 8.5-Mb interval, which includes the 2.14-Mb *CEN178* satellite repeat arrays (Fig. 3D) [22]. As noted, *CEN3* in Col and Ler contain adjacent inverted *CEN178* arrays (Figs. 1 and 3) [22]. The remainder of the *CTL3.9* interval is heterochromatic, containing numerous DNA and RNA transposable elements, yet shows increasing gene density towards the *CTL3.9* T-DNAs (Fig. 3E,F).

Arabidopsis T-DNAs may be present as multi-copy insertions, or associated with chromosome rearrangements, in addition to being DNA methylated [51, 52]. As both structural variation and DNA methylation suppress Arabidopsis crossovers [9, 47, 48], we sought to map the genetic and epigenetic state of the *CTL3.9* T-DNAs. We sequenced the *CTL3.9* line using Oxford Nanopore Technology (ONT) and performed genome assembly (Additional file 9: Fig. S6). The *CTL3.9* genome assembly showed no evidence of large-scale structural rearrangements, compared to the parental Col line (Additional file 9: Fig. S6A). The *CTL3.9* loci CG17 (green) and CR55 (red) comprise 12.7- and 5.1-kb insertions flanked by T-DNA border sequences (Additional file 9: Fig. S6C-S6D). We used Deepsignal-plant to map patterns of DNA methylation over the *CTL3.9* T-DNAs, using our ONT data [53]. The T-DNAs were DNA methylated in CG, CHG, and CHH sequence contexts, although at lower levels than flanking transposable elements (Additional file 9: Fig. S6E-S6F). Although DNA methylation and structural hemizygosity of the FTL T-DNAs may cause local crossover suppression, these effects are likely to be limited relative to the size of the entire ~8.5 Mb *CTL3.9* interval. Furthermore, inheritance of red and green fluorescence from *CTL3.9* hemizygotes conformed to the Mendelian expectation of ~3:1 color:non-color, which is further consistent with the absence of rearrangements, or significant transgene silencing (Additional files 10-11: Tables S4-S5).

To map crossovers within *CTL3.9*, we crossed to the genetically polymorphic accession Ler. In wild type inbreds (Col/Col), *CTL3.9* has a genetic distance of 16.5 cM, equivalent to a recombination rate of 1.94 cM/Mb (Fig. 3B and Additional file 10: Table S4), compared to the genome average of 3.03 cM/Mb. In Col/Ler F_1_ hybrids, *CTL3.9* crossover frequency significantly increased relative to Col/Col inbreds, with a genetic distance of 18.3 cM, equivalent to 2.15 cM/Mb (Wilcoxon test *P=*2.74⨉10^−6^) (Additional file 11: Table S5). This is consistent with Arabidopsis hybrids showing higher crossover frequency than inbreds in other genetic intervals [54, 55]. From *CTL3.9* hemizygous Col/Ler F_1_ plants, we selected progeny seed that showed either red or green fluorescence alone, consistent with a single crossover between the T-DNAs (Fig. 3A). We selected 444 red-alone and 464 green-alone seeds, which were sown and genotyped with an array of 94 Kompetitive Allele-Specific PCR (KASP) markers for Col/Ler SNPs within *CTL3.9*, with an average inter-marker distance of 89.5 kb (Fig. 3A and Additional file 12: Table S6). We mapped 913 crossover events in total within *CTL3.9* (Additional file 13: Table S7). The genotypes of 908 plants were consistent with a single chromatid having one crossover and the other chromatid being non-recombinant, while two plants each contained two single crossover chromatids (Additional file 14: Fig. S7).

Crossovers were unevenly distributed within *CTL3.9*, and the recombination landscape was significantly correlated with crossovers mapped via whole-genome sequencing (*r*=0.719 *P*≤2.2⨉10^−16^) (Fig. 3B,C and Additional file 13: Table S7). To define crossover hotspots and coldspots, we calculated the expected number of events per interval, assuming an even distribution, and compared this to observed values (Additional file 15: Table S8). Observed and expected crossover counts for each interval were used in chi-square tests, followed by Bonferroni correction for multiple testing, to identify intervals that contained significantly higher or lower recombination. This approach identified four cold spot intervals (*CS1-CS4*), which occupy the central region of *CTL3.9*, and ten hotspots (*HS1-HS10*), which are distributed throughout the LRZs, with crossover rates in the range 10.8–19.5 cM/Mb (Fig. 3C and Additional file 15: Table S8). Several hotspots, for example *HS6* and *HS7*, are located inside dense heterochromatin, close to the NRZ boundaries (Fig. 3C). The *HS* intervals showed crossover rates comparable to previously mapped hotspots in the Arabidopsis genome [56–61]. We observed that compared to the coldspots, the hotspot intervals had significantly lower CG, CHG, and CHH context DNA methylation, ASY1, CENH3, H3K9me2, and REC ChIP-seq enrichment, and significantly higher H3K4me3 and H2A.Z ChIP-seq enrichment, and SPO11-1-oligos (all Wilcox tests <7.99⨉10^−3^) (Additional file 16: Fig. S8). We further correlated chromatin states with cM/Mb in all *CTL3.9* map intervals and observed significant negative relationships with DNA methylation (CG, CHG, and CHH contexts), H3K9me2, REC8, and ASY1 ChIP-seq, and positive relationships with SPO11-1-oligos, H3K4me3, and H2A.Z ChIP-seq (Fig. 3F-G). Overall, this is consistent with euchromatic marks promoting meiotic DSBs and crossovers, and heterochromatic marks repressing crossovers.

**Fig. 4:**
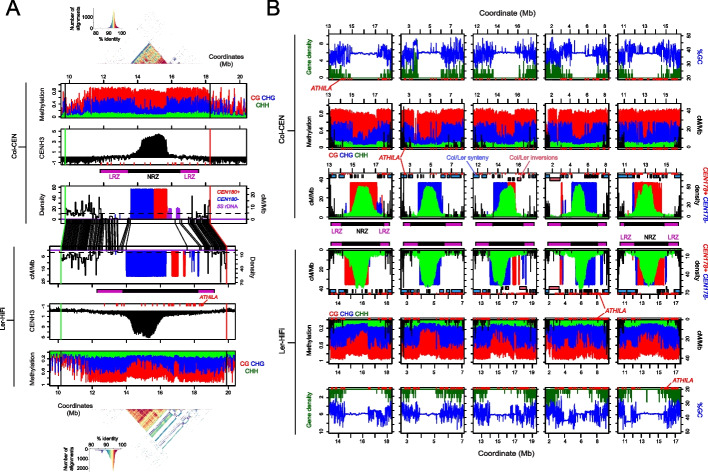
Genetic and epigenetic haplotypes of Col and Ler and centromere-proximal crossover recombination. **A** *CTL3.9* crossover frequency (cM/Mb) is plotted against the Col-CEN assembly, overlaid with a plot of the density of *CEN178* satellite repeats (red=forward, blue=reverse strand) and *5S* rDNA (purple). The positions of the *CTL3.9* fluorescent T-DNAs are shown by red and green vertical lines. Above this plot, the purple and black bars indicate the positions of the LRZs and NRZ. Above is a plot of CENH3 ChIP-seq enrichment (black), with *ATHILA* retrotransposons indicated by red *x*-axis ticks. Above this is a plot of the proportion of ONT-based DNA methylation in CG (red), CHG (blue), and CHH (green) sequence contexts. A StainedGlass sequence identity heat map is shown above the *CEN178* satellite arrays, together with a histogram indicating the color scale associated with % identity values. Beneath, a mirrored version is shown with data projected and aligned to the Ler-0 genome assembly. In the center of the plot, the physical positions of KASP and T-DNA markers in the Col-CEN and Ler-HiFi assemblies are connected with lines between the X-axis. **B** In the first row, the density of genes (green) is plotted alongside %GC content (blue) per 10 kb along the centromere-proximal regions of the Col-CEN genome assembly. *ATHILA *retrotransposon insertions are indicated as red *x*-axis ticks. In the next row, the proportion of DNA methylation mapped from ONT reads is plotted in 10-kb windows for CG (red), CHG (blue), and CHH (green) sequence contexts. In the next row, Col/Ler crossover frequency (cM/Mb) is plotted in 10-kb windows (black), with *CEN178* satellite density (red=forward & blue=reverse strand), and CENH3 ChIP-seq enrichment (green, log_2_[ChIP/input]) plotted in the same windows. The locations of non-recombining zones (NRZ, black), and low-recombining zones (LRZ, purple), are indicated beneath. Shaded blocks at the top of these plots indicate regions of Col/Ler synteny (blue) and inversions (pink) mapped by SyRI [37]. The lower three rows are the same as above, but for the Ler genome assembly. Information on chromatin datasets analyzed is available in Additional file 6: Table S2

To explore how genetic and epigenetic polymorphism influence recombination, we projected our *CTL3.9* crossover data onto the Col-CEN and Ler-HiFi genome assemblies (Fig. 4A). The LRZs and NRZ show structural polymorphism (Fig. 4A), which may directly contribute to crossover suppression in the central cold spot intervals. We compared epigenetic maps of DNA methylation and CENH3 ChIP-seq enrichment generated for each Col and Ler genome assembly (Fig. 4A) [21]. The Col and Ler centromeres both contain ~1-Mb regions of CENH3 ChIP-enrichment within the center of the *CEN178* arrays, which are typified by reduced CHG context DNA methylation relative to the flanking pericentromeres (Fig. 4A). Similarly, we compared the distributions of mapped crossovers in proximity to the Col and Ler centromere NRZs and LRZs, in relation to *CEN178* satellites, CENH3 occupancy, DNA methylation, gene density, and %GC content (Fig. 4B). In all cases, the NRZs are structurally polymorphic and contain the CENH3-occupied *CEN178* satellite arrays and show relatively high GC base content and CG context DNA methylation. This analysis is further consistent with a combination of DNA sequence variation and chromatin factors determining centromere-proximal recombination in Arabidopsis. Although we have mapped CENH3 enrichment and DNA methylation in the homozygous Col and Ler parental strains, we cannot exclude that distributions may differ in the Col/Ler F_1_ hybrids from which crossovers were mapped.

### The centromere-proximal *HOTSPOT6* contains gene islands embedded in heterochromatin

**Fig. 5:**
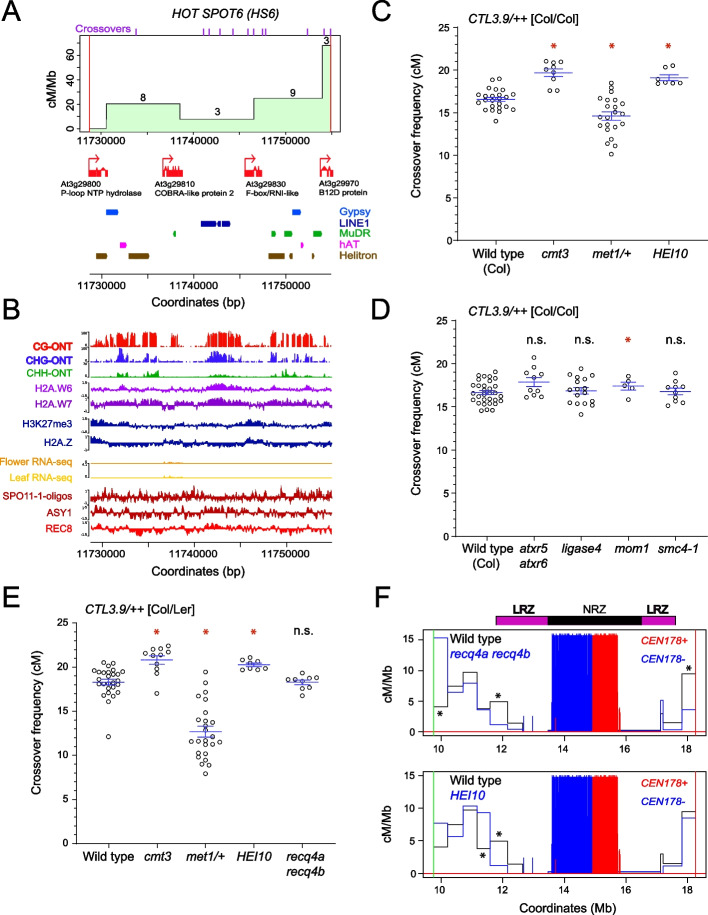
Genetic and epigenetic control of crossover frequency within *CTL3.9* and the *HS6* hot spot. **A** Derived cleaved amplified polymorphic sequence (dCAPS) markers were used to map crossovers within *HOTSPOT6* (*HS6*) and calculate cM/Mb. The numbers printed above the plot line show the number of crossovers identified in each interval. The midpoints of crossovers mapped by sequencing, as in Fig. 1, are indicated as purple ticks along the top axis. Gene annotation (red) is shown underneath, in addition to Gypsy/Ty3 (blue), LINE1 (dark blue), MuDR (green), hAT (pink), and Helitron (brown) transposon annotations. **B** Plots of the *HS6* interval showing ONT-derived DNA methylation (%) in CG (red), CHG (blue), or CHH (green) sequence contexts, ChIP-seq enrichment (log_2_(ChIP/input)) for H2A.W6 (light purple), H2A.W7 (dark purple), H3K27me3 (blue), H2A.Z (blue), ASY1 (dark red), and REC8 (red), RNA-seq from floral (orange) and leaf (yellow) tissue, and SPO11-1-oligos (dark red). Information on chromatin datasets analyzed is available in Additional file 6: Table S2. **C** *CTL3.9* crossover frequency (cM) in wild type, *cmt3*, *met1-3/+*, and *HEI10*, in an otherwise Col/Col homozygous background. Measurements from individuals are shown as circles. Horizontal blue lines represent the mean and the standard error of the mean. Red stars indicate samples which are significantly different from wild type using Wilcoxon tests, whereas “n.s.” indicates a non-significant difference. **D** As for **C**, but analyzing wild type, *atxr5 atxr6*, *ligaseIV*, *mom1*, and *smc4-1* mutants in a Col/Col homozygous background. **E** As for **C**, but with genotypes in a Col/Ler hybrid background, and with the addition of *recq4a recq4b*. **F** Comparison of crossover frequency (cM/Mb) in wild type, *recq4a recq4b* and *HEI10*, generated by SSLP mapping in Col/Ler F_2_ populations. The position of the *CTL3.9* T-DNAs is indicated by green and red vertical lines. The density of *CEN178* on the forward (red) and reverse (blue) strands are plotted. Crossover counts per interval were compared between genotypes using chi-square tests. Asterisks indicate intervals that had significantly different counts between wild type and *HEI10* or *recq4a recq4b*

*HOTSPOT6* (*HS6*) is centromere-proximal on the left arm and showed an elevated crossover rate (17.9 cM/Mb) (Fig. 3C). We sought to fine-map *HS6* crossovers (*n*=23) using an additional four Col/Ler-derived Cleaved Amplified Polymorphic Sequence (dCAPS) markers within *HS6*, and genotyped each of the 23 samples that contained a crossover (Fig. 5A and Additional file 17: Table S9). Crossovers were observed throughout *HS6*, with highest rates towards the centromere-proximal boundary (Fig. 5 and Additional file 17: Table S9). Four genes are located within *HS6*, encoding a *P*-loop NTP hydrolase, COBRA-like2, an F-box protein, and a B12D transmembrane protein (Fig. 5B), which are conserved between the Col and Ler assemblies (Additional file 18: Fig. S9). Of these genes, *COBRA-like2* showed highest evidence of expression from RNA-seq data (Fig. 5B) [45]. The *HS6* genes are either methylated in the CG context, or unmethylated, whereas the intervening regions contain multiple transposon sequences that are densely DNA methylated in CG and non-CG sequence contexts (Fig. 5B). The gene regions were also distinguished by higher H3K27me3 and H2A.Z ChIP-seq enrichment (Fig. 5B) [40]. SPO11-1-oligos within *HS6* were denser in proximity to the genes and reduced within the heterochromatic repeats, whereas the opposite was true for REC8-cohesin and ASY1 ChIP-seq (Fig. 5B) [28, 41]. This shows that *HS6* crossovers are associated with gene islands that are otherwise embedded in repetitive heterochromatin.

### Genetic control of crossover frequency within *CENTROMERE3*

To investigate genetic control of centromere-proximal crossovers, we crossed *CTL3.9* to mutants with changed recombination or chromatin pathways, in inbred (Col/Col) backgrounds. The mutants used were (i) *chromomethylase3* (*cmt3-11*), which is required for maintenance of CHG DNA methylation [62], (ii) *methyltransferase1* (*met1*), which is deficient in maintenance of CG DNA methylation [63], (iii) *atxr5 atxr6*, which are required for maintenance of the heterochromatic mark H3K27me1 [29], (iv) *smc4-1*, which disrupts a condensin complex required for gene silencing and chromosome compaction [64], (v) *mom1*, which is defective in DNA-methylation-independent gene silencing and regulates transcription of *CEN178* repeats [65], (vi) *HEI10*, which over-expresses a pro-crossover E3 ligase [36], (vii) *ligase4*, which is required for nonhomologous end joining (NHEJ) and DSB repair in repetitive regions [66], and (viii) *recq4a recq4b*, which is deficient in DNA helicases that negatively regulate crossover frequency [67].

We observed that *cmt3-11* caused a significant increase in *CTL3.9* crossover frequency (Wilcoxon test *P*=6.60⨉10^−4^) (Fig. 5C and Additional file 10: Table S4), consistent with previous observations [47]. *CTL3.9* was crossed to generate *met1-3/+* heterozygous plants, which have reduced crossovers comparable to *met1-3* homozygotes yet have higher fertility [48]. *CTL3.9* crossover frequency was significantly reduced in *met1-3/+* (Wilcoxon test *P*=0.048) (Fig. 5C and Additional file 10: Table S4). Hence, CG and CHG context DNA methylation maintenance have antagonistic effects on centromere-proximal crossover rate. No significant change in *CTL3.9* crossover frequency occurred in *smc4*, *atxr5 atxr6*, or *lig4*, and a weak yet significant difference was observed in *mom1* (Wilcoxon test *P*=0.029) (Fig. 5D and Additional file 19: Table S10).

In Arabidopsis, crossovers are generated by Class I and Class II pathways, which differ in their sensitivity to interference and interhomolog genetic polymorphism [54, 55, 68]. Therefore, we investigated *CTL3.9 *crossovers in wild type, *cmt3*, *met1/+*, *HEI10*, and *recq4 recq4b* Col/Ler F_1_ hybrids, to compare with the respective inbred backgrounds (Fig. 5C–E and Additional file 11: Table S5). Increased and decreased *CTL3.9* crossover frequencies were replicated in *cmt3* (Wilcoxon test *P*=5.80⨉10^−3^) and *met1/+* hybrids (Wilcoxon test *P*=1.12⨉10^−9^), showing that these changes are insensitive to interhomolog polymorphism (Fig. 5E and Additional file 11: Table S5). The *recq4a recq4b* mutant causes increased Class II crossovers in the chromosome arms, whereas *HEI10* overexpression increases the Class I pathway [32, 36, 69]. Despite strong effects on recombination in the chromosome arms [32, 67], *recq4a recq4b* did not significantly change *CTL3.9* crossovers, whereas *HEI10* showed a relatively weak but significant increase (Wilcoxon test *P*=5.55⨉10^−4^) (Fig. 5E and Additional file 11: Table S5).

Although overall crossover rate was not dramatically increased, we performed further genetic mapping within *CTL3.9* to determine if spatial crossover patterns were changed in *HEI10* or *recq4a recq4b* hybrids. We genotyped 90 crossovers in wild type, 92 in *recq4a recq4b* and 90 in *HEI10* using ten Simple Sequence Length Polymorphism (SSLP) markers distributed throughout *CTL3.9* (Fig. 5F and Additional file 20: Table S11). In *recq4a recq4b*, crossover frequency significantly increased in the interval closest to the green T-DNA insertion, and decreased next to the red T-DNA, and at the left distal LRZ border (chi-square tests *P*≤0.05) (Fig. 5F and Additional file 20: Table S11). In *HEI10*, a significant crossover change was observed close to the left distal LRZ border (chi-square test *P*≤0.05) (Fig. 5F and Additional file 20: Table S11). Although significant changes were observed in a minority of intervals, *HEI10* and *recq4a recq4b* had relatively limited effect on the *CTL3.9* recombination rate and landscape.

### The centromere-proximal recombination landscape is remodelled in *cmt3* and *met1/+* DNA methylation mutants

**Fig. 6:**
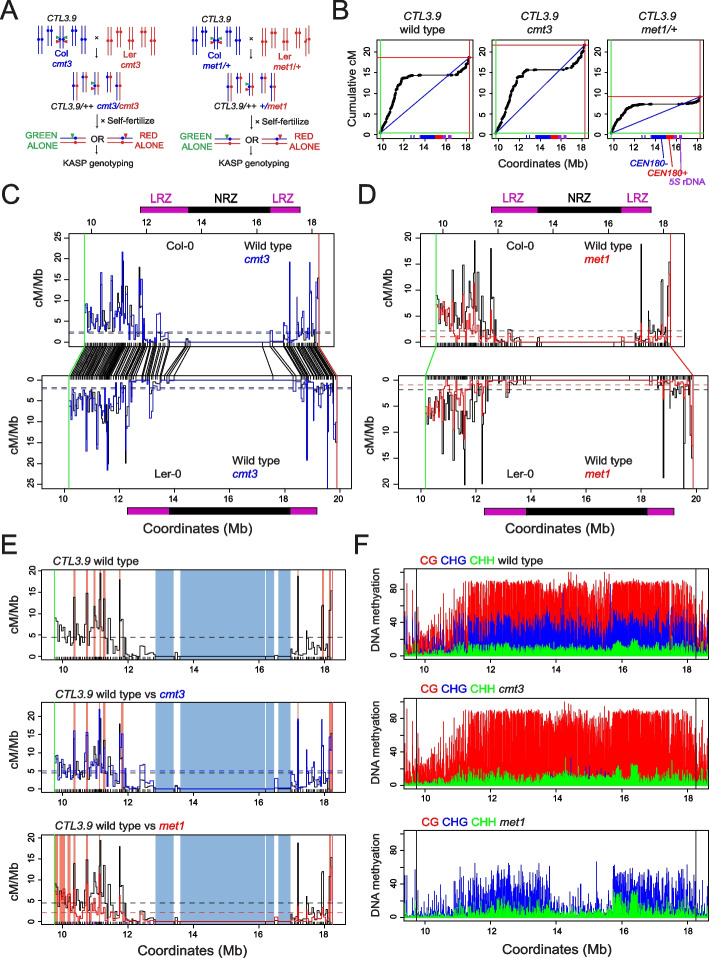
Remodelling of the centromere-proximal crossover landscape in wild type, *cmt3*, and *met1/+*. **A** Genetic strategy to recover crossovers within the *CTL3.9* centromeric FTL interval from *cmt3* and *met1/+* mutants. FTL T-DNAs encoding red and green fluorescent proteins are indicated by triangles. The parental chromosomes are from the Col (blue) and Ler (red) accessions. **B** Cumulative genetic map (centiMorgans, cM) relative to *CTL3.9* genomic coordinates against the Col-CEN assembly in wild type, *cmt3*, and *met1/+*. The blue diagonal lines show a linear relationship, with the red and green vertical/horizontal lines showing the location of the *CTL3.9* T-DNAs. The position of *CEN178* satellite repeats are shown as red (forward) and blue (reverse) ticks on the *x*-axis, in addition to *5S* rDNA (purple). **C** Plots of crossover frequency (centiMorgans per Megabase, cM/Mb) within *CTL3.9* in wild type (black) and *cmt3* (red) projected against the Col (upper) or Ler (lower) assemblies, with mean values shown by dotted horizontal lines. The position of KASP genotyping markers are indicated as black *x*-axis ticks, and *CTL3.9* T-DNAs are indicated by red and green lines, and connected between the maps by lines. The LRZs (purple) and NRZ (black) as defined in wild type are shown as colored blocks above and below the plots. **D** As for **C**, but plotting wild type (black) and *met1/+* (red) crossover frequency (cM/Mb). **E** *CTL3.9* crossover frequency (cM/Mb) in wild type (upper), *cmt3* (middle), and *met1/+* (lower), with significantly high (hot spots, pink) and low (cold spots, blue) intervals shown by colored shading. **F** Plots of DNA methylation (%) across *CTL3.9*derived from BS-seq data in wild type, *cmt3*, and met1 [70, 71], colored according to CG (red), CHG (blue) and CHH (green) sequence contexts. The position of the flanking *CTL3.9* T-DNAs are indicated by black vertical lines. Information on DNA methylation datasets analyzed is available in Additional file 6: Table S2

As DNA methylation maintenance mutants changed *CTL3.9* crossover frequency in a hybrid background, we sought to perform high-resolution recombination mapping in *cmt3* and *met1/+*. Using the same fluorescent selection approach as for wild type, we mapped 1033 crossovers within *CTL3.9* from *cmt3*, and 962 from *met1/+* (Fig. 6A and Additional file 13: Table S7)*.* The crossover landscape was significantly correlated between wild type, *cmt3*, and *met1/+* (wt vs *cmt3 r=*0.743, wt vs *met1 r*=0.852) (Fig. 6B–D and Additional file 13: Table S7). To test for regional recombination changes within *CTL3.9*, we tallied crossovers in the NRZ, LRZs, and distal regions and compared wild type, *cmt3*, and *met1/+*. The LRZs showed significantly higher crossovers in *cmt3* (18.3%, chi test *P*=2.23×10^−5^), and significantly lower crossovers in *met1/+* (7.0%, chi test *P*=1.17×10^−3^), compared to wild type (11.4%) (Fig. 6B–D and Additional file 13: Table S7). In contrast, the distal regions significantly decreased in *cmt3* (81.6%, chi test *P*=2.82×10^−5^) and increased in *met1* (93.0%, chi test *P*=1.17×10^−3^), compared to wild type (88.6%) (Fig. 6B–D and Additional file 13:Table S7). Only a single NRZ crossover was observed in *cmt3*, indicating that the NRZ are stably repressed for crossover across these genotypes. Together this shows that relative crossover distributions have changed within *CTL3.9* between wild type, *cmt3*, and *met1/+*.

We tested for significantly hot or cold intervals in *met1/+* and *cmt3* compared to the random expectation. This identified that the large central *CS* cold spots maintained crossover suppression in both mutants (Fig. 6E and Additional files 13 and 15: Tables S7-S8). The *cmt3* mutant showed fewer hotspots (*n*=8) than wild type (*n*=10), six of which were in the same locations (Fig. 6E and Additional files 13 and 15: Tables S7-S8). In *met1/+*, the fine-scale landscape was more significantly changed compared to wild type, with hotspots in proximity to the *CEN178* arrays being strongly suppressed (Fig. 6D,E and Additional files 13 and 15: Tables S7-S8). For example, *HS6* and *HS7* are no longer significant hotspots in *met1/+*. Instead, new *met1/+* hotspots are detected close to the distal boundaries of the interval, with nine *met1/+* hotspots observed that were not present in wild type, together with five that were shared between genotypes (Fig. 6E and Additional files 13 and 15: Tables S7-S8). Together, this shows distinct patterns of remodelling of the crossover landscape within *CTL3.9* in *cmt3* and *met1/+*.

The changes in *cmt3* crossover distribution correlates with a strong reduction in CHG context DNA methylation within the centromere and pericentromere (Fig. 6F) [22, 70, 71]. Hence, loss of CHG methylation increases centromere-proximal crossover frequency, but the overall topology of the recombination landscape remains similar to wild type. The *met1* mutant shows a strong reduction in CG context DNA methylation across the centromere region, in addition to depletion of non-CG methylation within the *CEN178* arrays (Fig. 6F) [22, 70, 71]. These changes to the *met1* centromeric DNA methylation landscape associate with a reduction and redistribution of crossovers, which was distinct from the crossover changes observed in *cmt3*. This demonstrates the contrasting effects of CG and CHG context DNA methylation maintenance on the centromere-proximal recombination landscape in Arabidopsis.

## Discussion

Despite playing a conserved function in kinetochore complex assembly, the size, DNA sequence, and structure of eukaryotic centromeres are divergent within and between species [19–21]. Although centromeres are diverse at the DNA sequence level, they also share the feature of suppressed meiotic crossovers across species [8, 10, 12, 22]. It has been proposed that suppression of centromere-proximal crossovers reduces gamete aneuploidy [8, 14, 72]. Consistently, we observed that crossovers are completely absent within the centromere NRZs, which contain the *CEN178* satellite arrays and regions of CENH3 enrichment, implying that NRZ crossovers may also be deleterious in Arabidopsis. Additionally, we identified 1–2 Mb pericentromeric LRZs that surround the NRZs and experience strongly suppressed crossovers. The low levels of crossovers that occur within the LRZs are locally enriched within isolated euchromatic gene islands.

We propose that centromeric crossover suppression is caused by a combination of NRZ structural polymorphism and repressive heterochromatic marks, including dense DNA methylation, H3K9me2, and CENH3. We propose that centromere-proximal crossover suppression arises as (i) the number of DSB precursors occur at lower levels [22, 42, 47], and that (ii) heterochromatin and (iii) structural polymorphism further inhibit downstream crossover repair pathways. A fourth possibility is that the centromere and kinetochore complex actively recruit factors that suppress crossover repair or that the kinetochore might impose physical constraints that suppress crossover formation. As the centromeric and pericentromeric regions are enriched for REC8-cohesin [28], by analogy with budding yeast this may promote meiotic DSB repair using a sister chromatid, contributing to crossover suppression [8, 73]. Meiotic DSBs could also be channelled into non-crossover repair [2], thereby limiting centromere-proximal crossovers. In comparison, human centromeres are composed of similar megabase-scale α-satellite arrays, although narrower (~100 kb) regions of CENP-A enrichment are observed, which are DNA hypomethylated in the CG sequence context [23, 25]. As human centromeres are also suppressed for meiotic crossovers [10], species-specific chromatin and DNA sequence organization may play varying roles in shaping centromere-proximal recombination landscapes.

We demonstrate that changes to the DNA methylation landscape, via mutation of either the CG (*met1/+*) or CHG (*cmt3*) maintenance pathways, cause contrasting changes to the landscape of centromeric crossover frequency. In wild type, a zone of crossover suppression extends from the NRZ into the flanking LRZs. In *met1/+* mutants, the zone of LRZ crossover suppression appears to extend and causes clustering of new recombination hotspots in more distal locations. This effect is reminiscent of the long-range effects of crossover interference [34, 74], and we propose that a *cis*-acting recombination-suppressing signal emanating from the centromere may be strengthened in *met1*. In contrast, loss of CHG DNA methylation in *cmt3* increases LRZ crossovers, at the expense of the distal regions, although the NRZ remains strongly crossover-suppressed. In *Drosophila melanogaster*, crossovers are strongly suppressed in the centromere cores, in addition to the flanking pericentromeric heterochromatin [75]. Interestingly, in *Drosophila Blm* mutants (Blm is a RECQ4 ortholog), the centromere-core remains crossover-suppressed, but the flanking low-recombining zones increase recombination, despite normal levels of heterochromatin [75]. As the *Blm* mutant disrupts multiple aspects of crossover patterning in *Drosophila* [76], this is further consistent with centromere-proximal suppression of recombination involving long-range communication.

Previous work revealed that both *met1* and *cmt3* experience greater levels of pericentromeric meiotic DSBs, measured by SPO11-1-oligo sequencing [42, 47]. Hence, divergent changes occur to the centromere-proximal crossover landscape in these mutants, despite similar increases to the initiating DSBs [42, 47]. The *met1* and *cmt3* mutants are distinguished in at least two ways at the chromatin level; (i) H3K9me2 is largely intact in *met1*, but is reduced in *cmt3* [71, 77], and (ii) in *cmt3*, CG and CHH methylation are intact, but CHG is very reduced, whereas in *met1* CG is globally reduced and CHG/CHH methylation are also reduced within the *CEN178* arrays [22, 70, 71]. We propose that differences in DNA methylation context between *met1* and *cmt3*, and associated chromatin marks, have varying effects on meiotic recombination repair, downstream of SPO11-1-dependent DSBs. Further profiling of meiotic recombination, including single-strand invasion, joint molecule formation and crossover resolution in these mutants may thus be revealing.

We observed a significant number of genes in the LRZs and NRZs that showed evidence of gene expression from RNA-seq data. Consistent with population genetics expectations for crossover-suppressed regions [78], these genes have significantly higher levels of genetic diversity than in the chromosome arms. As crossovers occur at low levels in the centromere-proximal regions, mutations in these genes may not be efficiently purged via selection and therefore may accumulate over time, although other evolutionary forces, including varying mutation rates, may also contribute to the observed higher genetic diversity. LRZ/NRZ genes experience similar levels of SPO11-1-oligo formation in their promoters and terminators, compared with genes located in the chromosome arms. As the LRZ/NRZ genes show elevated levels of the heterochromatic marks DNA methylation and H3K9me2, we propose that despite initiation of meiotic recombination, downstream crossover repair is inhibited. The LRZs and NRZs are also strongly enriched for Gypsy/Ty3 retrotransposons, which may reflect integration bias, or post-insertion effects of selection removing insertions in the chromosome arms. One consequence of gene and transposon co-location within the NRZs/LRZs is that they will tend to maintain linkage with each other and the centromere. The extent to which centromere-proximal recombination suppression is important for chromosome segregation will be interesting to explore, in addition to how linked-inheritance influences genetic variation and evolution of genes and transposons that reside in these regions.

## Conclusion

Our work shows the genetic and epigenetic organization of the *A. thaliana* centromeres and flanking pericentromeric heterochromatin and how this relates to the zones of crossover suppression that surround the CENH3-occupied satellite repeat arrays. We conclude that centromere-proximal crossover suppression is caused by a combination of structural genetic polymorphism and epigenetic chromatin states, including DNA methylation and CENH3.

## Methods

### Plant material and growth

*Arabidopsis thaliana recq4a-4* (GABI_203C07), *recq4b-2* (N511330/N660303) [79], *cmt3-11* (N648381) [80], *met1-3* (N16394) [63], *atxr5* (N630607), *atxr6* (N866134) [29], *mom1* (N826153) [65], *ligaseIV* (N656431) [66], and *smc4-1* (N69854) [64] mutants, the traffic line *CTL3.9* [50], and the *HEI10* over-expressor line [36], were generated in the Col-0 background. The *met1-3* allele was backcrossed into Ler [48], and the *cmt3-7*(N6365) and *recq4a-W387X* (EMS) [67] mutants are in the Ler accession [62]. Plants were grown in controlled environment chambers at 20°C under long day conditions (16/8 h light/dark photoperiods) with 60% humidity and 150 μmol m^−2^ s^−1^ light intensity. To stratify germination, once seeds were sown on commercial soil, they were kept for 2 days in the dark at 4°C before transferring them into growth chambers. Genotyping primers are provided in Additional file 21: Table S12.

### Mapping crossovers from sequencing data and NRZ and LRZ identification

Whole-genome Illumina resequencing datasets of Arabidopsis Col (SRX202246) and Ler (SRX202247) were downloaded from the NCBI SRA database [81]. The paired-end raw reads were evaluated for quality using FastQC (version 0.11.9), and then adapter and low-quality sequences were trimmed using Trimmomatic (version 0.38) [82]. BWA (version 0.7.15-r1140) was used to align short reads with default parameters to the Col-CEN reference genome [22, 83] (https://github.com/schatzlab/Col-CEN). Read alignments with a mapping quality greater than 20 were considered as uniquely mapped and used in subsequent analysis. Tandem repeat finder (version 4.09) was used with default parameters to scan the centromere-masked genome for tandem repeats, and used for quality examination of proximal SNPs [84].

To obtain high-confidence SNPs that can differentiate Col and Ler genotypes, we used the following strategy. First, SNPs and structural variants (SVs) were predicted from the sequencing datasets of Col and Ler accessions using inGAP-family [85]. Then, SNPs were filtered to remove potential false positives that arise from sequencing errors, small indels, tandem repeats, and SVs using inGAP-family, as described previously [85, 86]. Further, the SNPs were evaluated and filtered in both BC_1_ and F_2_ populations. Whole-genome Illumina resequencing datasets of Col/Ler BC_1_ (E-MTAB-11254) and F_2_ (E-MTAB-8165) populations were downloaded from the ArrayExpress database at EMBL-EBI and aligned to Col-CEN, as above [9, 33]. For the BC_1_ population data, SNPs were retained if (i) the Col allelic ratio (Col read number divided by total read number) was larger than 0.6 and less than 0.9, and (ii) the homozygous Col allelic ratio (number of samples with 0/0 genotype divided by the total number of genotyped samples) was larger than 0.4 and less than 0.7, and (iii) the heterozygous Col allelic ratio (number of samples with 0/1 genotype divided by total number of genotyped samples) was larger than 0.3 and less than 0.6, and (iv) the homozygous Ler allelic ratio (number of samples with 1/1 genotype divided by total number of genotyped samples) was less than 0.1, and (v) the total number of genotyped samples was larger than 70 (5-quantiles). For the F_2_ population, SNPs were retained if (i) the Col allelic ratio was larger than 0.3 and less than 0.7, and (ii) the homozygous and heterozygous Col, and the homozygous Ler allelic ratios were between 0.1 and 0.9, and (iii) the total number of genotyped samples was larger than 5. Following both BC_1_ and F_1_ filtering, a set of 334,680 high-quality Col/Ler SNPs were retained for subsequent analysis.

For each BC_1_ and F_2_ sample, the read count and genotype profile of the filtered high-quality SNPs were produced by inGAP-family, and then a sliding window-based method was adopted for detecting crossovers, using a window of 70 kb, and a step size of 35 kb [33–35]. Final crossover positions were further refined by examining the genotype information of individual proximal SNPs. Poorly covered (<0.1× depth) and potentially contaminated samples (>2% of windows with first-allele frequency in the range of 0.8 to 0.9) were removed from further analysis. To further filter false crossovers caused by mis-genotyping, we calculated the double-crossover frequency, defined as the number of samples with double crossovers divided by the number of samples with crossovers in the given window, for every 1-Mb window, with a 500 kb step size. We filtered double crossovers in the given window, when the double-crossover frequency was greater than 4%. Moreover, we manually examined crossovers that were located close to each side of the centromere of each chromosome for the BC_1_ and F_2_ populations, respectively. To be retained, a qualified crossover had to be supported by sufficient coverage of Col and Ler-specific reads, with a minimum of five accumulated reads, in both homozygous and heterozygous genotypes. A total of 1,009, 978, and 12,410 crossovers were retained for the female BC_1_, male BC_1_, and F_2_ populations, respectively. Crossovers were then tallied in 100-kb windows along the Col-CEN genome. The non-recombining zones (NRZs) were defined as the contiguous regions containing the main *CEN178* arrays where crossovers were not observed. From the boundaries of the NRZs, we defined the LRZs, as the 1-cM flanking regions. The 14,397 mapped crossovers were also tallied in the *CTL3.9* KASP marker windows to correlate recombination rates between experiments.

To identify crossover locations against the Ler-HiFi assembly, we extracted 2 kb of sequence from the Col-CEN assembly around the midpoint of the 14,397 crossover locations mapped against the Col-CEN genome. These sequences were used to perform alignments against the Ler-HiFi assembly using LASTZ [39]. Of the 14,397 crossovers, four could not be aligned to the Ler assembly using this method. For each crossover, we selected the alignment with highest % alignment coverage for subsequent analysis. We removed crossovers that aligned to multiple locations with equally high % coverage or % identity values, which reduced the crossovers to a set of 13,718 high-confidence events. We then calculated NRZ and LRZ coordinates in the same way as for the Col-CEN assembly.

### Genome analysis and annotation

The Col-CEN genome was used for analysis [21, 22]. The Ler assembly is from ENA study ID PRJEB55353 and corresponds to ENA assembly ID GCA_946406525 [21]. Gene, transposon, and tandem repeat annotation for the Col and Ler genome assemblies were as reported [21, 22]. StainedGlass (version 0.5) was used to generate sequence identity heat maps, using a 10,000 bp window size, to compare the repeat architecture of Col and Ler centromeres [38]. Minimap2 (version 2.24) and SyRI (version 1.6) were used to map regions of synteny and inversions between the Col and Ler assemblies [37, 87]. To map the *CTL3.9* recombination data onto the Ler assembly, the sequences surrounding the KASP SNPs were aligned to Ler-0 using LASTZ (version 1.04.15) [39].

### Analysis of genes and transposons in the LRZs and NRZs

Using the LRZ and NRZ coordinates against the Col-CEN assembly, we identified contained genes. These genes were masked for those located within the mitochondrial genome insertions on chromosome 2, and those located in the large pericentric Col/Ler inversion on chromosome 4 [43]. Fine-scale profiles around genes located in the LRZs (*n*=542), NRZs (*n*=132), or the chromosome arms (*n*=27,499), or the same number of randomly positioned loci of the same number and width distribution within the same regions, were calculated for ChIP-seq, RNA-seq, and bisulfite-seq data sets by providing igwig files to the computeMatrix tool from deepTools (version 3.1.3) in “scale-regions” mode [88]. Each feature was divided into non-overlapping, proportionally scaled windows between start and end coordinates, and flanking regions were divided into 10-bp windows. Mean values for each data set were calculated within each window, generating a matrix of profiles in which each row represents a feature with flanking regions and each column a window. Coverage profiles for an input sequencing library and a gDNA library were used in conjunction with those for ChIP-seq and SPO11-1-oligo libraries, respectively, to calculate windowed log_2_([ChIP+1]/[control+1]) coverage ratios for each feature. Meta-profiles (windowed means and 95% confidence intervals) for each group of features were calculated and plotted using the feature profiles in R (version 4.0.0). Information on all chromatin datasets analyzed is available in Additional file 6: Table S2.

We tested crossovers for overlap with LRZ genes using permutation tests, which compared the observed number of gene-overlapping crossovers with the numbers of gene-overlapping randomly positioned LRZ loci, across 10,000 permuted sets of the same number and widths as the crossovers. An equivalent procedure was performed to test for overlap of LRZ crossovers with transposon annotation. The length (kb) of transposon annotation was also calculated in 100-kb windows and plotted along the Col-CEN assembly for the Gypsy/Ty3, Copia/Ty1 and LINE superfamilies*.*

To investigate genetic diversity in LRZ and NRZ genes, permutation analysis of pairwise diversity (Pi, *π*) in genes was performed. We downloaded the variant call format (VCF) and annotation of the 1135 A. thaliana natural accessions from Phytozome (https://phytozome-next.jgi.doe.gov) [46]. We calculated pairwise diversity (*π*) for TAIR10 gene models, allowing for 15% missing calls per site. We removed sites that overlapped transposable elements, rDNA, plastid sequences, and simple repeats in TAIR10 from the VCF file. We required that at least half of the length of the gene had sequencing coverage among the 1135 accessions, in order to produce a reliable *π* calculation per gene. We excluded genes that overlapped inversions between the Col-CEN and Ler-HiFi genome assemblies, in addition to genes that overlapped the mitochondrial insertion on chromosome 2 from the analysis. After filtering 336 LRZ and 58 NRZ genes were retained for analysis. We calculated median *π* for this filtered gene set separately for the NRZ and LRZ genes, and compared this value to 1000 permutations of median *π* for genes in the chromosome arms.

### Assembly of the *CTL3.9* FTL line genome and comparison with Col-CEN

Oxford Nanopore sequencing of homozygous *CTL3.9* plants was performed as reported [22]. Reads were trimmed of adapter sequences using Porechop (version 0.2.4) and filtered for length and accuracy using Filtlong (version 0.2.0) (--min_mean_q 90, --min_length 30000). The trimmed and filtered reads were assembled using Flye (version 2.7) [89]. Flye contigs were scaffolded and orientated using RagTag (version 2.0.1) using the reference genome Col-CEN. To compare the CTL3.9 and Col-CEN assemblies, sequence identity dot plots were performed using ReDOTable (https://www.bioinformatics.babraham.ac.uk/projects/redotable/). T-DNA borders were identified by using LASTZ to search for the T-DNA left border sequence. EDTA was used to annotate transposons in the CTL3.9 assembly [90]. DNA methylation in the CTL3.9 genome was mapped using the ONT reads and Deepsignal-plant, as described [22]. The CG17 and CR55 T-DNAs were identified using the primers as listed in Table S11. Based on alignments of the T-DNAs to the *CTL3.9* genome assembly, we observed two T-DNA insertions in tandem at CG17. In comparison to the Col-CEN assembly, 17 bp in the *CTL3.9* assembly is missing close to the CG17 T-DNA insertion site. Likewise, the CR55 T-DNA insertions are in tandem. Moreover, a 1289-bp region on one side of the CR55 T-DNA is duplicated on both sides of the insertion, which includes one gene (At3g06765) that encodes a non-coding RNA.

### Crossover measurement using *CTL3.9* seed fluorescence

*CTL3.9* comprises genetically linked T-DNAs expressing red or green fluorescent proteins in the seed from the *NapA* promoter that flank centromere 3 [50], which were used to measure crossover frequency and map recombination events. For each sample, three seed images were acquired; (i) brightfield, (ii) UV through a dsRed filter, and (iii) UV through a GFP filter, using a Fluorescent Stereomicroscope (Leica M165FC) [55]. CellProfiler (version 2.1.1) image analysis software was used to identify seed boundaries in micrograph images, and to assign RFP and GFP fluorescence intensity values to each seed object [55, 91]. In a *CTL3.9 RG/++* hemizygous line, when a single crossover occurs between the T-DNAs, they are inherited separately through meiosis, resulting in seed with red or green fluorescence alone. *CTL3.9* genetic distance can then be calculated using the formula;

$$\textrm{centiMorgans}\ \left(\textrm{cM}\right)=100\times \left(1-{\left[1-2\left({\textrm{N}}_{\textrm{G}}+{\textrm{N}}_{\textrm{R}}\right)/{\textrm{N}}_{\textrm{T}}\right]}^{1/2}\right)$$

where *N*_G_ is a number of green-alone fluorescent seeds, *N*_R_ is a number of red-alone fluorescent seeds, and *N*_T_ is the total number of seeds counted [55]. Statistical comparisons between samples were performed comparing the mean cM of replicate plants using Wilcoxon tests.

### *CTL3.9* KASP genotyping, crossover identification, and analysis

Single-nucleotide polymorphisms (SNPs) between the Col and Ler accessions and 50 base pairs of flanking sequence on each side of the SNP were used to design Kompetitive Allele Specific PCR (KASP) markers (LGC, Hoddesdon, UK). KASP uses two allele-specific forward primers and one common reverse primer. The two allele-specific primers possess unique tail sequences that correspond to a FRET (fluorescence resonant energy transfer) cassette; one labelled with FAM and the other with HEX. KASP allows differentiation of two alleles via competitive binding of allele-specific primers. If the genotype is homozygous, then only FAM or HEX fluorescent signals are observed. If the genotype is heterozygous, then a combination of FAM and HEX fluorescent signals is observed. The majority of SNPs analyzed were located within genes, with the remainder located in intergenic regions.

F_2_ seeds showing green or red fluorescence alone were manually selected and grown on soil, alongside wild type and Col/Ler F_1_ heterozygote controls. Leaf tissue was collected when plants were 3 to 4 weeks old. LGC performed DNA extraction from leaf tissue and KASP genotyping. Raw fluorescence data for each marker were assessed for differentiation of genotypes and used to annotate markers in each sample as either Col/Col, Col/Ler, or Ler/Ler genotype. Each plant was expected to contain a single crossover event within the *CTL3.9* interval, identified by a genotype transition from Col/Ler to Ler/Ler in green-alone seeds, or Ler/Ler to Col/Ler in red-alone seeds (Additional file 14: Fig. S7A). Most plants showed an expected genotype transition associated with a single crossover (Additional file 14: Fig. S7A).

A minority of plants (*n*=29) showed multiple genotype transitions, for example Col/Col to Col/Ler to Ler/Ler, or Ler/Ler to Col/Ler to Col/Col (Additional file 14: Fig. S7B). These genotypes can be explained if homozygous red or green recombinant seeds were selected, in which case two crossovers on different chromatids are present (Additional file 14: Fig. S7B). For these samples, both independent crossovers were counted. Three plants were found to be entirely Col/Col genotype, which likely reflect seed contamination, and these samples were removed from analysis (Additional file 14: Fig. S7C). Two remaining plants showed genotype transitions including Col/Ler to Col/Col to Col/Ler to Ler/Ler, or Ler/Ler to Col/Ler to Col/Col to Col/Ler, which are consistent with one recombinant chromatid and another chromatid with a double-crossover event carrying an introgression from Col (Additional file 14: Fig. S7D). In these cases, only one crossover chromatid was counted.

Twenty-six plants showed a missing genotype at the crossover site, meaning the recombination event could not be unambiguously placed in one of two adjacent intervals. In these cases, the single crossover value was divided between the two marker intervals, in proportion to the unambiguous crossovers mapped within the same two intervals. For example, if interval A contained 11 crossovers, and interval B contained 9, then a value of 0.55 would be added to interval A, and 0.45 would be added to interval B, in order to place the ambiguous crossover. To define crossover hotspot and coldspot intervals, we calculated the expected number of crossovers per interval, assuming an even distribution, and compared this to observed events. Observed and expected crossover counts for each interval were used to perform chi-square tests, followed by Bonferroni correction for multiple testing, to identify intervals that contained significantly higher or lower crossovers. This process was repeated separately for wild type, *met1/+*, and *cmt3*.

### Simple Sequence Length Polymorphism (SSLP) marker design

To design SSLP genetic markers, chromosome 3 genomic DNA sequence from TAIR10 (Col) (GenBank CP002686.1) and Ath.Ler-0.MPIPZ.v1.0 (Ler) (GenBank LR215054.1) were used. Mauve sequence alignment was performed between the Col and Ler sequences using Geneious Prime software. Primers were designed flanking identified Col/Ler indel polymorphisms (Additional file 21: Table S12). BLAST alignment to all chromosomes of the Col and Ler assemblies was performed on primer sequences to assess their uniqueness within the genome. Genetic markers were validated using leaf genomic DNA extracted from Col, Ler, and F_1_ (Col/Ler) genotypes, in addition to no template controls.

### Profiling DNA methylation in using Oxford Nanopore sequencing

This was performed, as reported [21, 22]. Briefly, for genomic DNA extraction for Oxford Nanopore Technologies (ONT) sequencing, 3-week-old *A. thaliana* plants, grown on 1/2 MS media containing 1% sucrose, were placed in the dark for 48 h prior to harvesting. Approximately 10 g of tissue was used per 200 ml of MPD-Based Extraction Buffer pH 6.0 (MEB). Tissue was flash frozen and then ground in liquid nitrogen using a pestle and mortar and resuspended in 200 ml MEB. Ground tissue was thawed in MEB with frequent stirring. The homogenate was forced through 4 layers of Miracloth, and then filtered again through 4 layers of fresh Miracloth by gravity. Triton x-100 was added to a final concentration of 0.5% on ice, followed by incubation with agitation on ice for 30 min. The suspension was centrifuged at 800*g* at 4°C for 20 min. The supernatant was removed and the pellet resuspended using a paintbrush in 10 ml 2-methyl-2,4 pentanediol buffer pH 7.0 (MPDB). The suspension was centrifuged at 650*g* at 4°C for 20 min. The supernatant was removed and the pellet was washed with 10 ml of MPDB. Washing and centrifugation was repeated until the pellet appeared white, when it was resuspended in a minimal volume of MPDB. From this point onwards, all transfers were performed using wide-bore pipette tips. Five milliliters CTAB buffer was added to the nuclei pellet and mixed via gentle inversion, followed by incubation at 60°C until full lysis had occurred, taking between 30 min and 2 h. An equal volume of chloroform was added and incubated on a rocking platform, with a speed of 18 cycles per minute, for 30 min, followed by centrifugation at 3000 g for 10 min. An equal volume of phenol/chloroform/isoamyl alcohol (PCI, 25:24:1) was added to the lysate, followed by incubation on a rocking platform (18 cycles per minute) for 30 min. The lysate was centrifuged at 3,000 *g* for 10 min and the upper aqueous phase was transferred into a fresh tube. The PCI extraction was then repeated. The extraction was repeated using only chloroform. One-tenth volume of 3M Sodium Acetate was added to the lysate and mixed by gentle inversion. Two volumes of ice-cold ethanol were added and mixed by inversion. DNA was precipitated at -20^o^C for 48 h. The precipitated DNA was removed using a glass hook and washed three times in 70% ethanol. The DNA was dissolved in 120 μl of 10 mM Tris-HCl pH 8.5.

Approximately 5 μg of DNA was size selected (>30 kb) using the BluePippin System (Sage Science) and the 0.75% agarose gel cassette (BLF7510, Biozym), using Range mode and BP start set at 30 kb. Library preparation followed the ONT SQK-LSK109 protocol kit, using 1.2-1.5 μg of size-selected DNA in a volume of 48 μl. DNA was nick-repaired and end-prepped by the addition of 3.5 μl of NEBNext FFPE Buffer and NEBNext Ultra II End Prep Reaction Buffer, followed by 2 μl of NEBNext DNA Repair Mix and 3 μl NEBNext Ultra II End Prep Enzyme Mix (New England Biolab, E7180S), with incubation for 30 min at 20°C, followed by 30 min at 65°C. The sample was cleaned using 1×volume AMPure XP beads and eluted in 61 μl of nuclease-free water. Adapters were ligated at room temperature using 25 μl Ligation Buffer, 10 μl NEBNext T4 DNA Ligase and 5 μl Adapter Mix for 2 h. The library was cleaned with 0.4×volume AMPure XP beads, washed using ONT Long Fragment buffer and eluted in 15 μl elution buffer.

We quantified CG, CHG, and CHH context DNA methylation with DeepSignal-plant (version 0.1.4), which uses a deep-learning method based on a bidirectional recurrent neural network (BRNN) with long short-term memory (LSTM) units to detect 5mC methylation. R9 reads were filtered for length and accuracy using Filtlong (Version 0.2.0) (--min_mean_q 90, --min_length 20000). Base-called read sequence was annotated onto corresponding .fast5 files, and re-squiggled using Tombo (version 1.5.1). Methylation prediction for the CG, CHG, and CHH contexts were called using DeepSignal-plant using the model: model.dp2.CNN.arabnrice2-1_120m_R9.4plus_tem.bn13_sn16.both_bilstm.epoch6.ckpt. The scripts “call_modification_frequency.py” and “split_freq_file_by_5mC_motif.py” provided in the DeepSignal-plant package were used to generate the methylation frequency at each CG, CHG, and CHH site.

### Profiling CENH3 in Ler-0 using ChIP-seq

This was performed, as reported [21, 22]. Briefly, approximately 12 g of 2-week-old seedlings were ground in liquid nitrogen. Nuclei were isolated in nuclei isolation buffer (1 M sucrose, 60 mM HEPES pH 8.0, 0.6% Triton X-100, 5 mM KCl, 5 mM MgCl2, 5 mM EDTA, 0.4 mM PMSF, 1 mM pepstatin-A and 1×protease inhibitor cocktail) and crosslinked in 1% formaldehyde at room temperature for 25 min. The crosslinking reaction was quenched with 125 mM glycine and incubated at room temperature for a further 25 min. The nuclei were purified from cellular debris via two rounds of filtration through one layer of Miracloth and centrifuged at 2500*g* at 4°C for 25 min. The nuclei pellet was resuspended in EB2 buffer (0.25 M sucrose, 1% Triton X-100, 10 mM Tris-HCl pH 8.0, 10 mM MgCl2, 1 mM EDTA, 5 mM DTT, 0.1 mM PMSF, 1 mM pepstatin-A and 1×protease inhibitor cocktail) and centrifuged at 14,000*g* at 4°C for 10 min. The nuclei pellet was resuspended in lysis buffer (50 mM Tris-HCl pH 8.0, 1% SDS, 10 mM EDTA, 0.1 mM PMSF and 1 mM pepstatin-A) and chromatin was sonicated using a Covaris E220 Evolution device with the following settings: power=150 V, bursts per cycle=200, duty factor=20% and time=90 s. Sonicated chromatin was centrifuged at 14,000*g* and the supernatant was extracted and diluted with 1×volume of ChIP dilution buffer (1.1% Triton X-100, 20 mM Tris-HCl pH 8.0, 167 mM NaCl, 1.1 mM EDTA, 1 mM pepstatin-A and 1×protease inhibitor cocktail). The chromatin was incubated overnight at 4°C with 50 μl Protein A magnetic beads (Dynabeads, Thermo Fisher) pre-bound with 2.5 μl α-CENH3 (gift of Prof. Steven Henikoff). The beads were collected on a magnetic rack and washed twice with low-salt wash buffer (150 mM NaCl, 0.1% SDS, 1% Triton X-100, 20 mM Tris-HCl pH 8.0, 2 mM EDTA, 0.4 mM PMSF, 1 mM pepstatin-A and 1×protease inhibitor cocktail) and twice with high-salt wash buffer (500 mM NaCl, 0.1% SDS, 1% Triton X-100, 20 mM Tris-HCl pH 8.0, 2 mM EDTA, 0.4 mM PMSF, 1 mM pepstatin-A and 1×protease inhibitor cocktail). Immunoprecipitated DNA–protein complexes were eluted from the beads (1% SDS and 0.1 M NaHCO_3_) at 65°C for 15 min. Samples were reverse crosslinked by incubating with 0.24 M NaCl at 65°C overnight. Proteins and RNA were digested with Proteinase K treatment, and RNase A, and DNA was purified by phenol:chloroform:isoamyl alcohol (25:24:1) extraction and ethanol precipitation. Library preparation followed the Tecan Ovation Ultralow System V2 library protocol. ChIP samples were PCR amplified for 12 cycles and sequenced with 150 bp paired-end reads on an Illumina instrument by Novogene.

Deduplicated paired-end CENH3 ChIP-seq Illumina reads (2×150 bp) from Col and Ler were processed with Cutadapt (version 1.18) to remove adapter sequences and low-quality bases (Phred+33-scaled quality <20). For each accession, trimmed reads were aligned to the respective genome assembly using Bowtie2 (version 2.3.4.3), using the following settings: --very-sensitive –no-mixed –no-discordant -k 10 –maxins 500. Up to 10 valid alignments were reported for each read pair. Read pairs with Bowtie2-assigned MAPQ <10 were discarded using Samtools (version 1.10). For retained read pairs that aligned to multiple locations, with varying alignment scores, the best alignment was selected. Alignments with more than 2 mismatches or consisting of only one read in a pair were discarded. For each data set, bins-per-million-mapped-reads (BPM; equivalent to transcripts-per-million, TPM, for RNA-seq data) coverage values were generated in igwig and bedGraph formats with the “bamCoverage” tool from deepTools (version 3.5.0). Reads that aligned to chloroplast or mitochondrial DNA were excluded from coverage normalization.

### Supplementary Information

**Additional file 1: Figure S1.** A refined pipeline for mapping crossovers from Col/Ler recombinant sequencing data.

**Additional file 2: Table S1.** Low-Recombining Zone and Non-Recombining Zone coordinates in the Col-CEN and Ler-HiFi assemblies. With reference to the Col-CEN assembly or the Ler-HiFi assembly, the chromosome length (bp) is given, in addition to the left and right low recombining zone (LRZ) and non-recombining zone (NRZ) boundaries. The start and end of the main *CEN178* satellite arrays are also given, for each chromosome.

**Additional file 3: Figure S2.** Crossover and SNP frequency plotted along the Ler genome assembly.

**Additional file 4: Figure S3.** Zones of centromeric crossover suppression and heterochromatic histone modifications.

**Additional file 5: Figure S4.** Zones of centromeric crossover suppression and REC8, ASY1 and SPO11-1-oligos.

**Additional file 6: Table S2.** Recombination and chromatin datasets analyzed in this study. This table provides references for each recombination and chromatin dataset analyzed in this study, which contain links to public databases where the data are available.

**Additional file 7: Figure S5.** Structural comparison of Col and Ler *CEN178* centromere satellite arrays.

**Additional file 8: Table S3.** Genes located within the LRZs and NRZs. The table provides whether genes are located in the LRZ or NRZ, their chromosome and start and end coordinate against the Col-CEN assembly, strand, gene name, TPM values for RNA-seq data generated from leaf or meiocytes [44], and gene annotation information from TAIR.

**Additional file 9: Figure S6.** Genetic and epigenetic structure of *CTL3.9* fluorescent crossover reporter T-DNAs.

**Additional file 10: Table S4.** *CTL3.9* fluorescent data and crossover frequency measurements in Col/Col inbred backgrounds. Crossover frequency measurements derived from fluorescent *CTL3.9/++* seed in wild type, *cmt3*, *met1/+* and *HEI10* overexpression lines. The “ctrl_1” samples are the control that was used for comparison with *met1/+* and *HEI10* overexpression lines, while “ctrl_2” were wild type controls for *cmt3*.

**Additional file 11: Table S5.** *CTL3.9* fluorescent data and crossover frequency measurements in Col/Ler hybrid backgrounds. Crossover frequency measurements derived from fluorescent *CTL3.9/++* seed in wild type, *cmt3, met1/+, HEI10* overexpression and *recq4a recq4b* lines. The “ctrl_1” is the control that was used for *recq4a recq4b*, and “ctrl_2” for *cmt3*, and “ctrl_3” for *met1/+* and *HEI10* overexpression lines.

**Additional file 12: Table S6.** Col/Ler SNPs used as markers for KASP genotyping within the *CTL3.9*. For each SNP, an id is assigned based on the TAIR10 coordinates of the SNP. IUPAC codes for the SNPs are indicated and the surrounding 50 bp region on either side of the SNP is shown. This data was provided to LGC (Hoddesdon, UK) to design KASP markers. The coordinates of SNPs in the TAIR10 and Col-CEN assemblies are provided.

**Additional file 13: Table S7.** *CTL3.9* crossover frequency maps in wild type, *met1/+* and *cmt3*. For each genetic interval with the *CTL3.9* map, the number of crossovers observed are shown for wild type, *cmt3* and *met1/+*, in addition to cM/Mb values.

**Additional file 14: Figure S7.** Genetic outcomes during *CTL3.9* fluorescent seed selection and genotyping.

**Additional file 15: Table S8.** Crossover hot spots and cold spots observed in the wild type, *cmt3* and *met1/+ CTL3.9* recombination maps. The table lists for the wild type, *cmt3* and *met1/+ CTL3.9*recombination maps, intervals that were significantly hot (hot spot, *HS*), or cold (cold spot, *CS*), based on observed and expected crossovers, assuming an even distribution. Observed and expected crossover counts for each interval were used to perform chi-square tests, followed by Bonferroni correction for multiple-testing, with the adjusted *P* value provided. Also provided are interval start coordinates and widths against the Col-CEN assembly, and the measured crossover rate (centiMorgans per Megabase, cM/Mb).

**Additional file 16: Figure S8.** Chromatin and recombination states within *CTL3.9* hotspots and coldspots.

**Additional file 17: Table S9.** Crossover frequency map in the *HS6* hotspot. 23 plants had a crossover event within the *HS6* hotspot. Recombinant plants were genotyped using four Col/Ler dCAPS markers (H6-1, H6-2, H6-3 and H6-4) to fine-map crossover locations.

**Additional file 18: Figure S9.** Structure of the *HOTSPOT6* genetic interval in the Col and Ler genome assemblies.

**Additional file 19: Table S10.** *CTL3.9* fluorescent data and crossover frequency measurements in wild type and *smc4, atxr5 atxr6, mom1, ligase IV* mutant backgrounds. Crossover frequency measurements derived from fluorescent *CTL3.9/++* seed in wild type, *smc4, atxr5 atxr6, mom1* and *ligaseIV* mutants. The “ctrl_1” samples are wild type controls for *smc4*, “ctrl_2” samples were wild type controls for *atxr5 atxr6*, “ctrl_3” samples were wild types for *mom1*, and “ctrl_4” samples were wild type controls for *ligase IV*.

**Additional file 20: Table S11.** *CTL3.9* SSLP genetic mapping in wild type, *recq4a recq4b* and *HEI10* overexpression lines. Crossover numbers, and cM/Mb values, identified within each interval using Col/Ler SSLP genotyping are shown in wild type, *recq4a recq4b* and *HEI10* overexpression.

**Additional file 21: Table S12.** Primer sequences used for *CTL3.9* SSLP and *HS6* dCAPs markers. The oligonucleotide sequences are provided that were used to; (i) validate T-DNA insertions, or the point mutations in various lines, (ii) to identify SSLPs to map *CEN3* proximal recombination events in wild type, *recq4a recq4b* and *HEI10* overexpression lines, (iii) to fine-map crossovers within *HOTSPOT6* in wild type as derived Cleaved Amplified Polymorphic Sequences (dCAPs) markers, and (iv) primers used to validate T-DNAs in CTL3.9 line.

**Additional file 22:.** Review history.

## Data Availability

Data generated or analyzed during this study are included in this published article and its supplementary information files. In addition, ONT sequencing reads (fastq format) and genome assembly (fasta format) of Arabidopsis transgenic line *CTL3.9* have been deposited in the Annotare repository (https://www.ebi.ac.uk/fg/annotare/) accession E-MTAB-13686 [92]. Computer R code used for data analysis is available from a GitHub page under the Creative Commons CC0-1.0 Universal licence (https://github.com/hendersi/Fernandes_et_al_scripts-data) [93] and at Zenodo: https://zenodo.org/doi/10.5281/zenodo.10429614 [94].
