# Supplementary material for: Structural variation and DNA methylation shape the centromere-proximal meiotic crossover landscape in Arabidopsis
Source: Genome Biol. 2024 Jan 22;25:30. doi: 10.1186/s13059-024-03163-4 (PMC10804481; doi:10.1186/s13059-024-03163-4)
Supplement: Supplementary file 1 — Additional file 1: Figure S1. A refined pipeline for mapping crossovers from Col/Ler recombinant sequencing data. [file 13059_2024_3163_MOESM1_ESM.pdf]

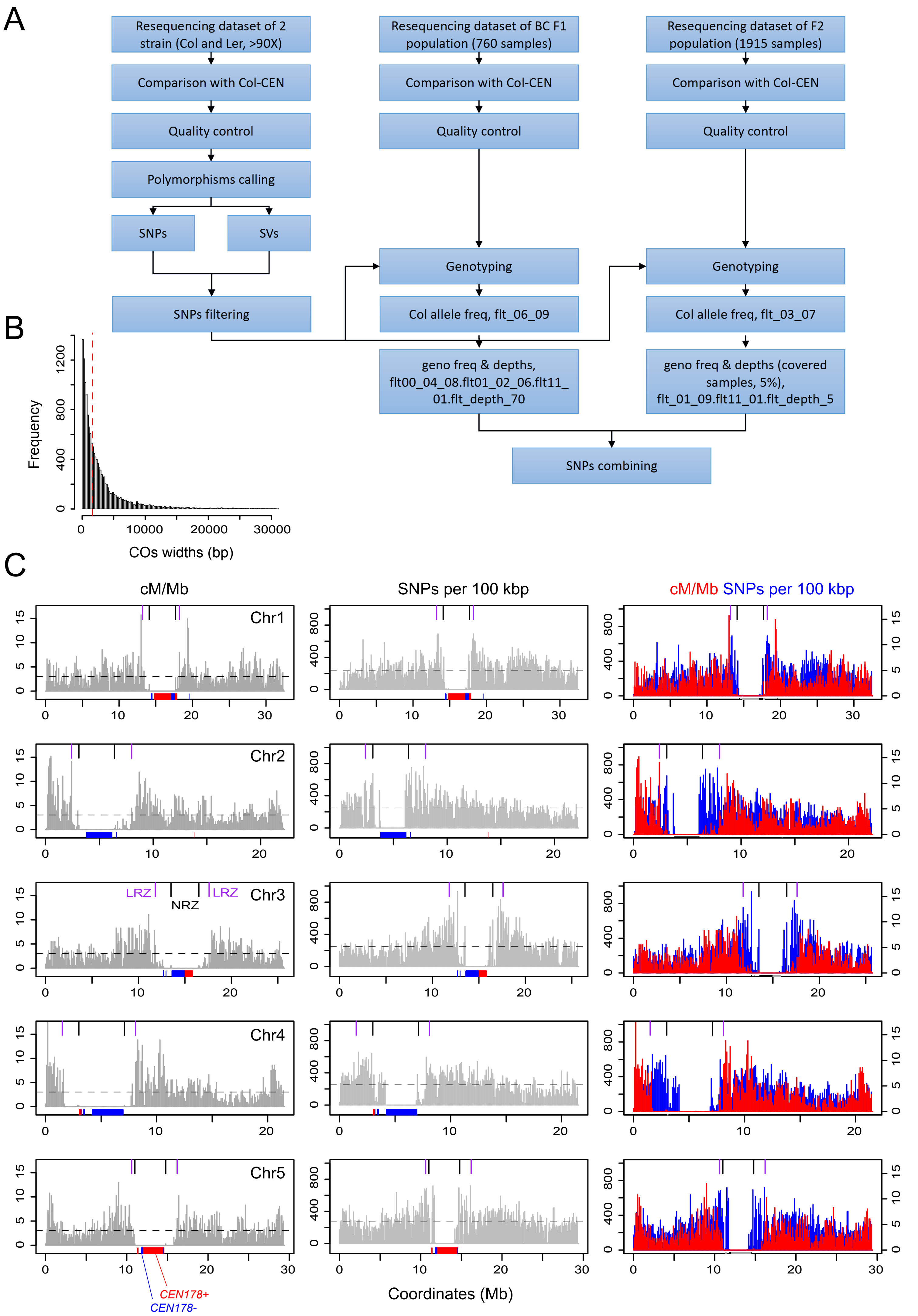
A
Resequencing dataset of 2 strain (Col and Ler, >90X)
Comparison with Col-CEN
Quality control
Polymorphisms calling
SNPs
SVs
SNPs filtering
Resequencing dataset of BC F1 population (760 samples)
Comparison with Col-CEN
Quality control
Genotyping
Col allele freq, flt_06_09
geno freq & depths, flt00_04_08.flt01_02_06.flt11_01.flt_depth_70
Resequencing dataset of F2 population (1915 samples)
Comparison with Col-CEN
Quality control
Genotyping
Col allele freq, flt_03_07
geno freq & depths (covered samples, 5%), flt_01_09.flt11_01.flt_depth_5
SNPs combining
B
Frequency
COs widths (bp)
C
cM/Mb
SNPs per 100 kbp
cM/Mb SNPs per 100 kbp
Chr1
Chr2
Chr3
Chr4
Chr5
LRZ
NRZ
LRZ
CEN178+
CEN178-
Coordinates (Mb)

Additional file 1: Figure S1. A refined pipeline for mapping crossovers from Col/Ler recombinant sequencing data. A. Schematic diagram showing the computational pipeline used to filter Col/Ler polymorphisms for SNPs that can be reliably used for crossover identification from backcross and F_2 populations. **B.** Histogram of crossover widths (bp) identified by our mapping pipeline, with the mean value shown by the dotted red line. **C.** 100 kb windows are plotted along the Col-CEN assembly showing crossover frequency (cM/Mb, left), SNPs (middle) and an overlay (cM/Mb=red, and SNPs=blue) along each chromosome. The horizontal dotted lines indicate genome average values. NRZ (black) and LRZ (purple) boundaries, as shown in Fig. 1, are indicated as ticks along the upper axis. *CEN178* positions are indicated as ticks along the x axis (red=forward strand, blue=reverse strand). Note that Col/Ler SNPs were identified between the genome assemblies using SyRI [41], which is not able to identify SNPs in highly repetitive and structurally polymorphic regions such as the centromeres.
