# Supplementary material for: Structural variation and DNA methylation shape the centromere-proximal meiotic crossover landscape in Arabidopsis
Source: Genome Biol. 2024 Jan 22;25:30. doi: 10.1186/s13059-024-03163-4 (PMC10804481; doi:10.1186/s13059-024-03163-4)
Supplement: Supplementary file 3 — Additional file 3: Figure S2. Crossover and SNP frequency plotted along the Ler genome assembly. [file 13059_2024_3163_MOESM3_ESM.pdf]

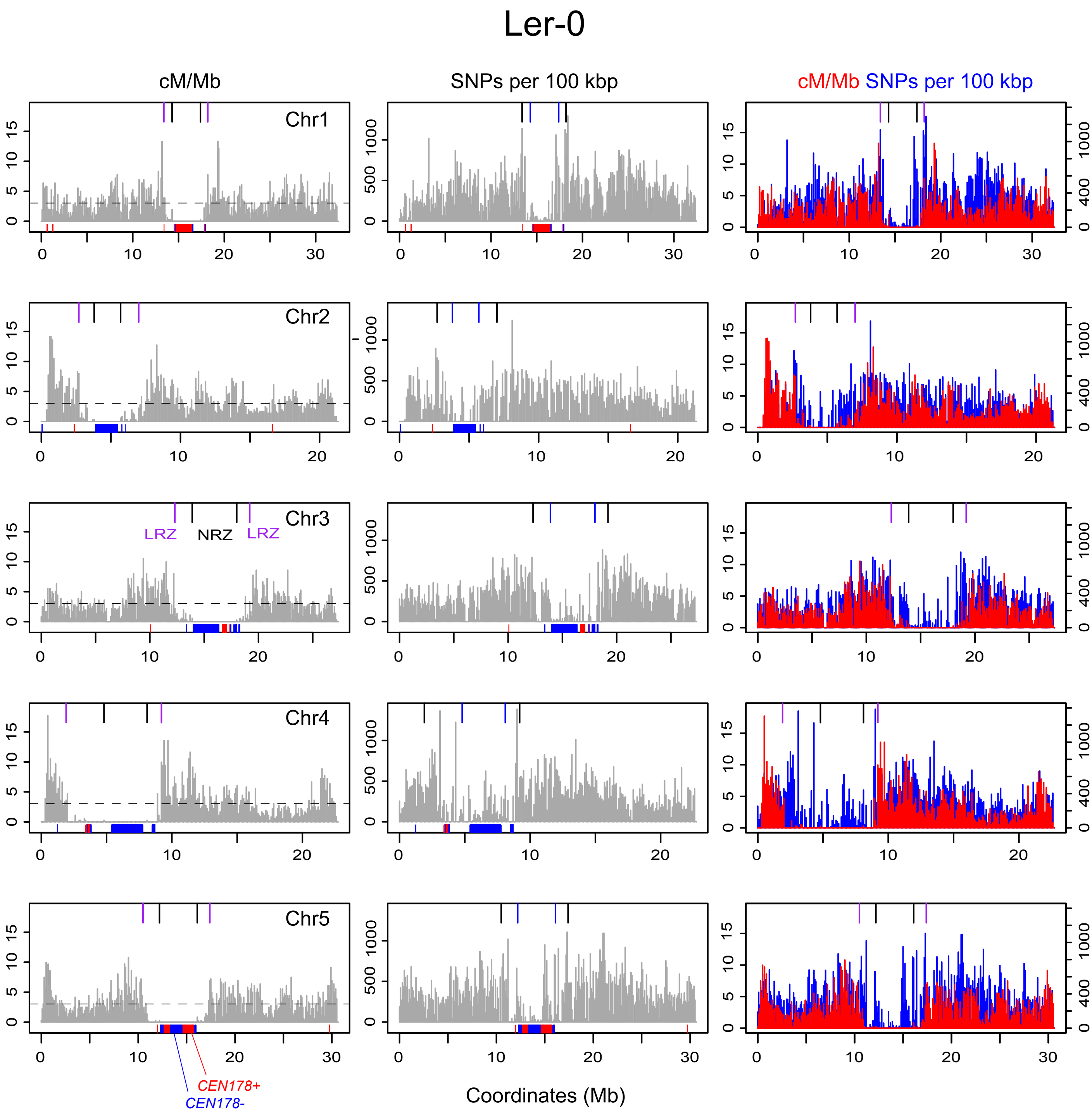
Ler-0
cM/Mb
SNPs per 100 kbp
cM/Mb SNPs per 100 kbp
Chr1
Chr2
Chr3
LRZ
NRZ
LRZ
Chr4
Chr5
CEN178+
CEN178-
Coordinates (Mb)

Additional file 3: Figure S2. Crossover and SNP frequency plotted along the Ler genome assembly. 100 kb windows are plotted along the Ler-HiFi assembly showing crossover frequency (cM/Mb, left), SNPs (middle), and an overlay (cM/Mb=red, and SNPs=blue) along each chromosome. The horizontal dotted lines indicate genome average values recombination rate. NRZ (black) and LRZ (purple) boundaries are indicated as ticks along the upper axis. Note that Col/Ler SNPs were identified between the genome assemblies using SyRI [41], which is not able to identify SNPs in structurally polymorphic regions, such as the centromeres. *CEN178* positions are indicated as ticks along the x axis (red=forward strand, blue=reverse strand).
