# Supplementary material for: Structural variation and DNA methylation shape the centromere-proximal meiotic crossover landscape in Arabidopsis
Source: Genome Biol. 2024 Jan 22;25:30. doi: 10.1186/s13059-024-03163-4 (PMC10804481; doi:10.1186/s13059-024-03163-4)
Supplement: Supplementary file 4 — Additional file 4: Figure S3. Zones of centromeric crossover suppression and heterochromatic histone modifications. [file 13059_2024_3163_MOESM4_ESM.pdf]

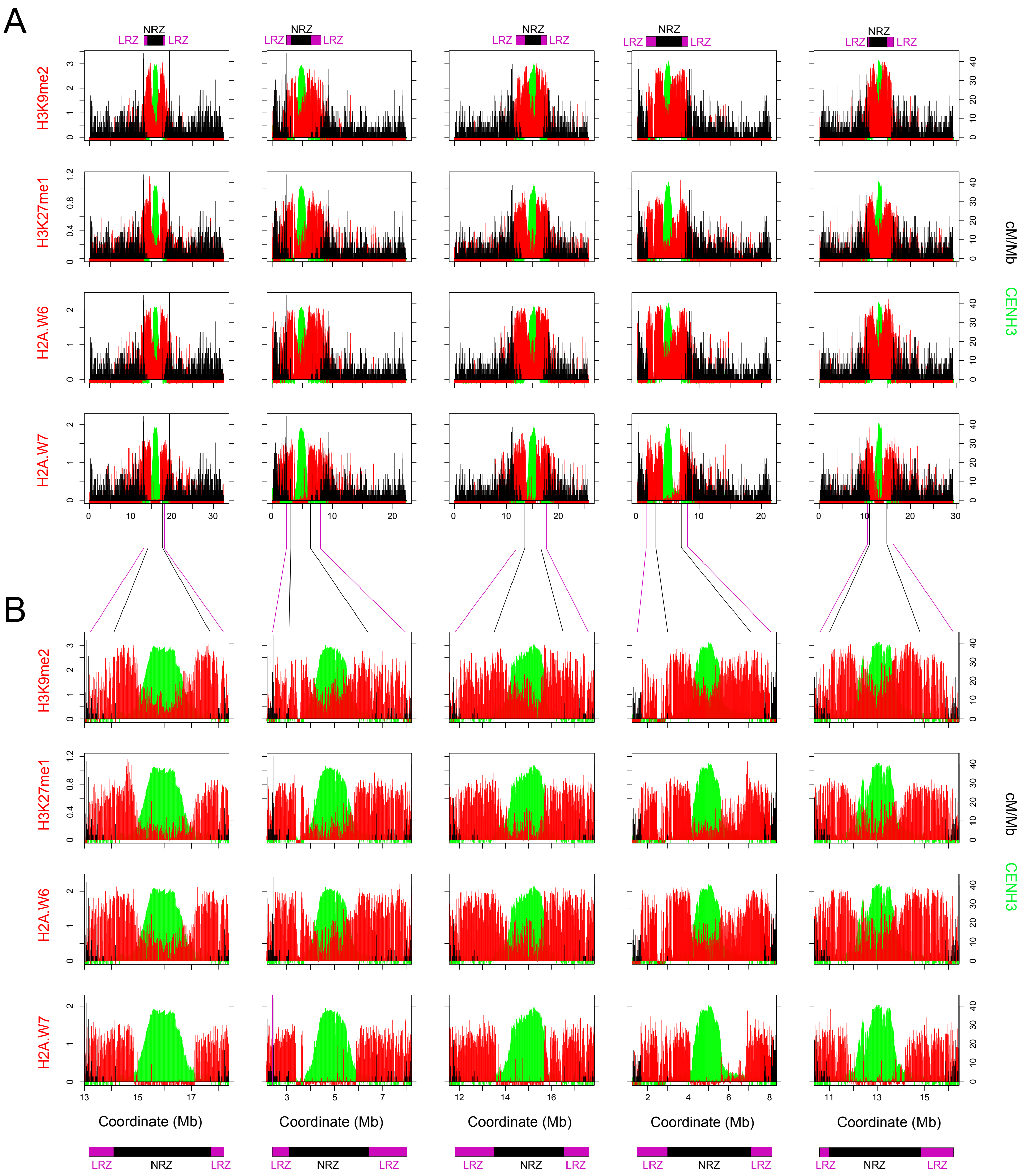
A
B
NRZ
LRZ
H3K9me2
H3K27me1
H2A.W6
H2A.W7
cM/Mb
CENH3
Coordinate (Mb)

Additional file 4: Figure S3. Zones of centromeric crossover suppression and heterochromatic histone modifications. A. Col/Ler crossover frequency (cM/Mb) mapped against the Col-CEN genome assembly in 10 kb windows is plotted (black). CENH3 ChIP-seq enrichment (green) is plotted using the same 10 kb windows. Above each plot, the location of the non-recombining zone (NRZ, black), and low-recombining zones (LRZ, purple), are indicated. Plots are shown compared to H3K9me2, H3K27me1, H2A.W6 and H2A.W7 ChIP-seq (red) enrichment for the same windows [28,29,38]. Information on chromatin datasets analysed is available in **Additional file 6: Table S2**. **B.** As for A, but showing a zoom of the NRZ and LRZ regions and the NRZ-LRZ positions are shown beneath by the black/purple bars.
