# Supplementary material for: Structural variation and DNA methylation shape the centromere-proximal meiotic crossover landscape in Arabidopsis
Source: Genome Biol. 2024 Jan 22;25:30. doi: 10.1186/s13059-024-03163-4 (PMC10804481; doi:10.1186/s13059-024-03163-4)
Supplement: Supplementary file 7 — Additional file 7: Figure S5. Structural comparison of Col and Ler CEN178 centromere satellite arrays. [file 13059_2024_3163_MOESM7_ESM.pdf]

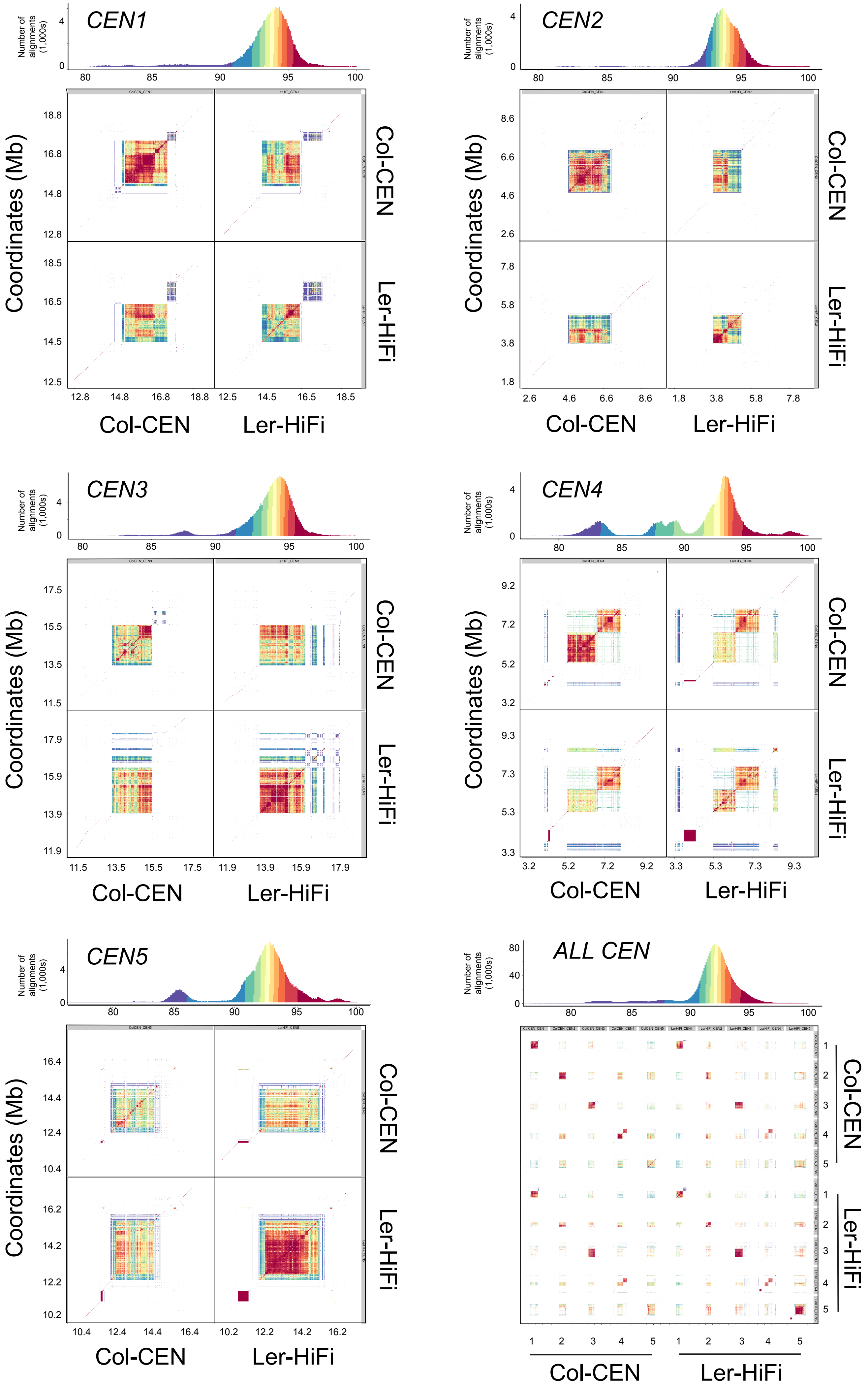
CEN1
Number of alignments (1,000s)
4
0
80
85
90
95
100
Coordinates (Mb)
18.8
16.8
14.8
12.8
18.5
16.5
14.5
12.5
12.8
14.8
16.8
18.8
12.5
14.5
16.5
18.5
Col-CEN
Ler-HiFi
CEN2
8.6
6.6
4.6
2.6
7.8
5.8
3.8
1.8
2.6
4.6
6.6
8.6
1.8
3.8
5.8
7.8
CEN3
17.5
15.5
13.5
11.5
17.9
15.9
13.9
11.9
11.5
13.5
15.5
17.5
11.9
13.9
15.9
17.9
CEN4
9.2
7.2
5.2
3.2
9.3
7.3
5.3
3.3
3.2
5.2
7.2
9.2
3.3
5.3
7.3
9.3
CEN5
16.4
14.4
12.4
10.4
16.2
14.2
12.2
10.2
10.4
12.4
14.4
16.4
10.2
12.2
14.2
16.2
ALL CEN
80
40
0
1
2
3
4
5

Additional file 7: Figure S5. Structural comparison of Col and Ler *CEN178* centromere satellite arrays. Sequence regions including and surrounding the main *CEN178* satellite arrays were compared between the Col and Ler assemblies, for each chromosome, using sequence identity heat maps generated by StainedGlass [85]. In addition, a comparison of all centromeres between Col and Ler is shown (lower right). A histogram of % sequence identity values within each set of heat maps is shown above, which indicates color correspondences. Regions shaded red show highest levels of pairwise sequence identity.
