# Supplementary material for: Structural variation and DNA methylation shape the centromere-proximal meiotic crossover landscape in Arabidopsis
Source: Genome Biol. 2024 Jan 22;25:30. doi: 10.1186/s13059-024-03163-4 (PMC10804481; doi:10.1186/s13059-024-03163-4)
Supplement: Supplementary file 9 — Additional file 9: Figure S6. Genetic and epigenetic structure of CTL3.9 fluorescent crossover reporter T-DNAs. [file 13059_2024_3163_MOESM9_ESM.pdf]

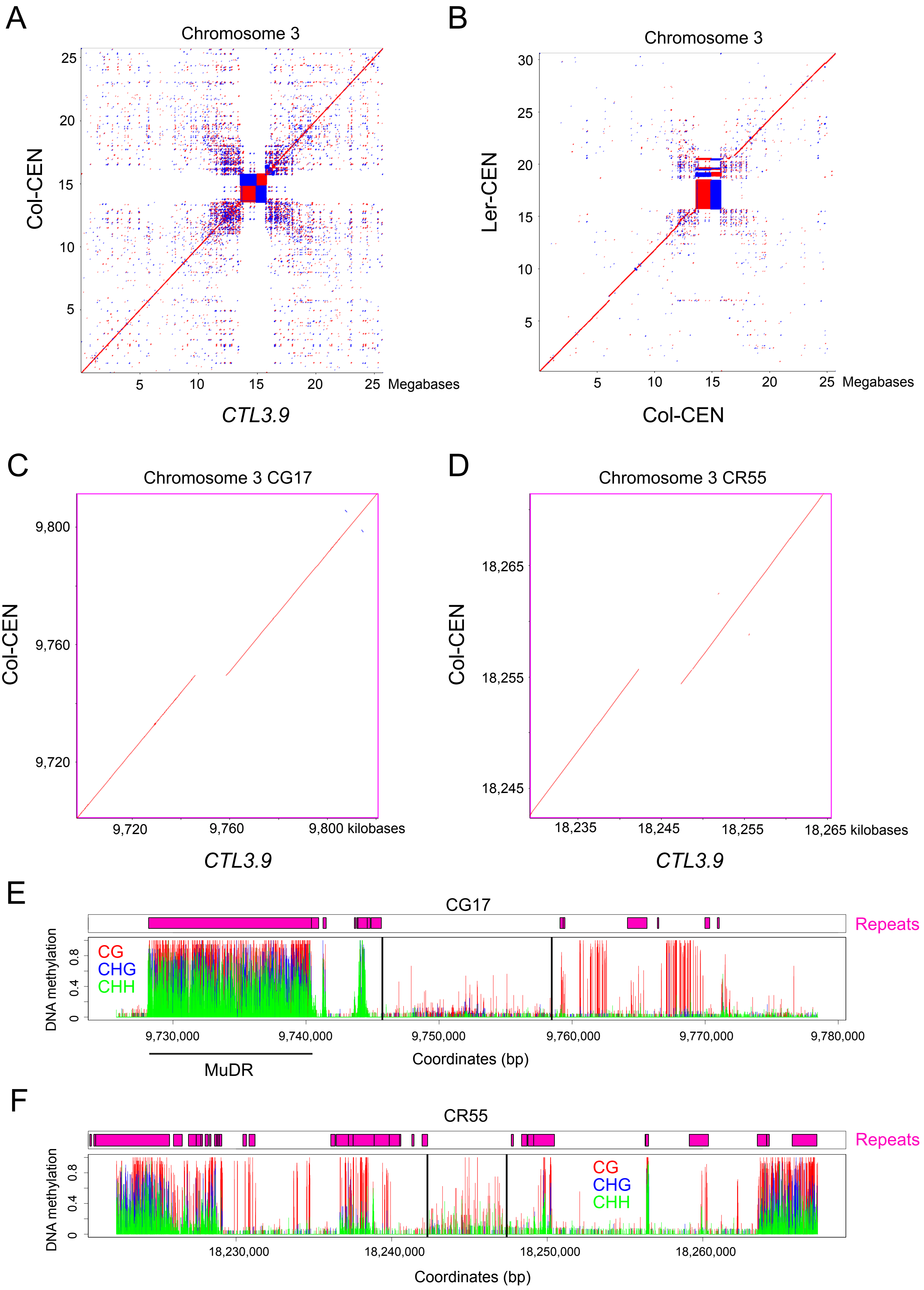

A
Chromosome 3
Col-CEN
CTL3.9
Megabases
B
Chromosome 3
Ler-CEN
Col-CEN
Megabases
C
Chromosome 3 CG17
Col-CEN
9,800
9,760
9,720
kilobases
CTL3.9
D
Chromosome 3 CR55
Col-CEN
18,265
18,255
18,245
18,235
kilobases
CTL3.9
E
CG17
Repeats
DNA methylation
CG
CHG
CHH
9,730,000
9,740,000
9,750,000
9,760,000
9,770,000
9,780,000
Coordinates (bp)
MuDR
F
CR55
Repeats
DNA methylation
CG
CHG
CHH
18,230,000
18,240,000
18,250,000
18,260,000
Coordinates (bp)

Additional file 9: Figure S6. Genetic and epigenetic structure of *CTL3.9* fluorescent crossover reporter T-DNAs. A. Dot plot analysis of chromosome 3 from the Col (Col-CEN) and *CTL3.9* genome assemblies generated using re-DOT-able (https://www.bioinformatics.babraham.ac.uk/projects/redotable/). Red and blue shading indicate forward and reverse strand similarity, respectively. **B.** As for A, but comparing chromosome 3 from the Col-CEN and Ler assemblies. **C.** As for A, but showing the region surrounding the CG17 *CTL3.9* T-DNA insertion. **D.** As for A, but showing the region surrounding the CR55 *CTL3.9* T-DNA insertion. **E.** ONT-based DNA methylation maps (proportion) for CG (red), CHG (blue) and CHH (green) sequence contexts across the CG17 T-DNA region. The T-DNA boundaries are indicated by vertical black lines. Above the plot, the pink rectangles indicate EDTA repetitive sequence annotation. **F.** As for E, but showing the region surrounding the CR55 T-DNA.
