# Supplementary material for: Structural variation and DNA methylation shape the centromere-proximal meiotic crossover landscape in Arabidopsis
Source: Genome Biol. 2024 Jan 22;25:30. doi: 10.1186/s13059-024-03163-4 (PMC10804481; doi:10.1186/s13059-024-03163-4)
Supplement: Supplementary file 14 — Additional file 14: Figure S7. Genetic outcomes during CTL3.9 fluorescent seed selection and genotyping. [file 13059_2024_3163_MOESM14_ESM.pdf]

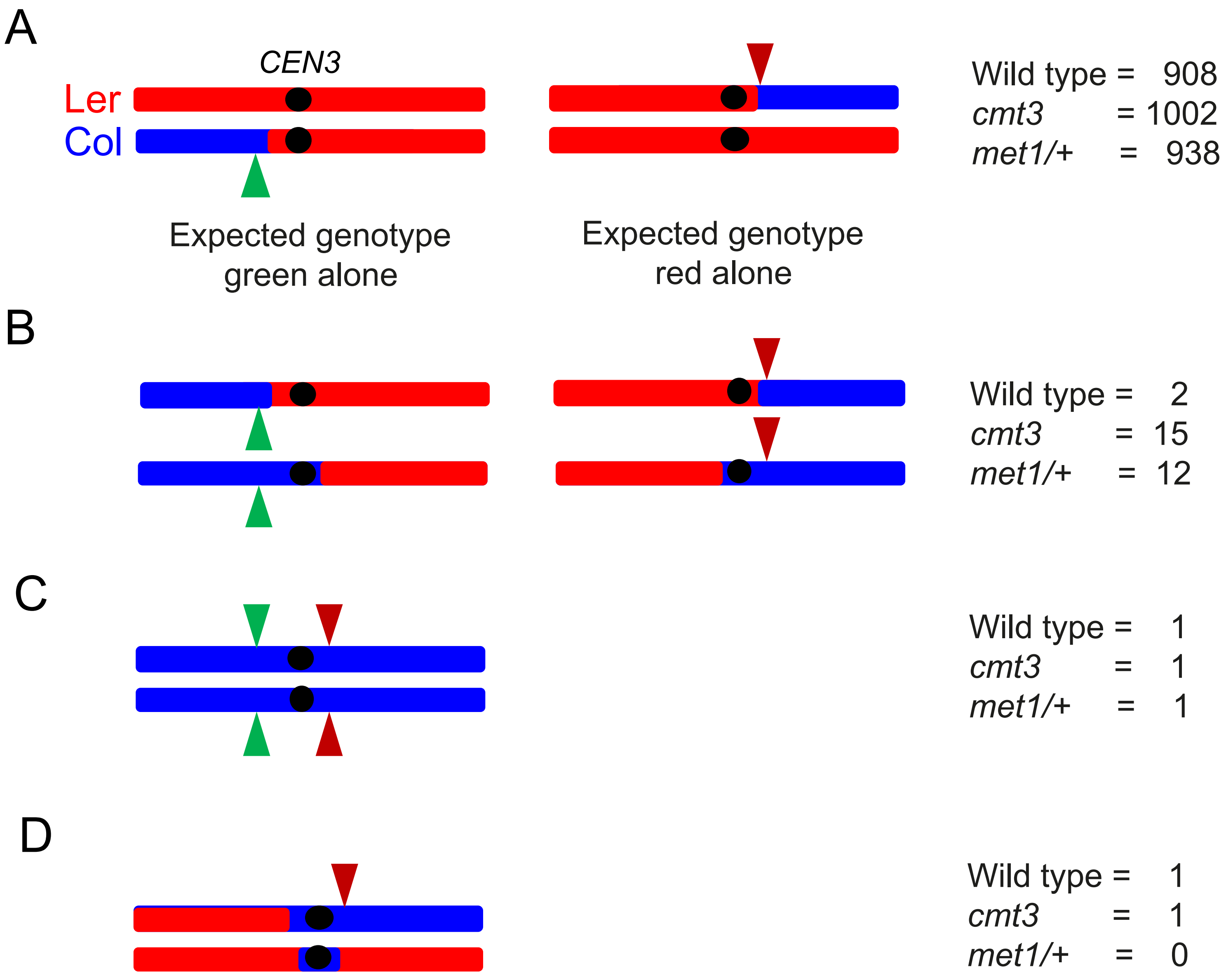

A
CEN3
Ler
Col
Expected genotype green alone
Expected genotype red alone
Wild type = 908
cmt3 = 1002
met1/+ = 938
B
Wild type = 2
cmt3 = 15
met1/+ = 12
C
Wild type = 1
cmt3 = 1
met1/+ = 1
D
Wild type = 1
cmt3 = 1
met1/+ = 0

Additional file 14: Figure S7. Genetic outcomes during *CTL3.9* fluorescent seed selection and genotyping. A. Diagrams showing the expected genotype of the majority of the seed selected as having green- or red-alone fluorescence, from a Col/Ler *CTL3.9*/++ hybrid. Red and blue indicate Ler and Col genotypes, respectively. The position of centromere 3 (*CEN3*) is indicated with a black circle. The number of plants showing this genotype pattern for each population are shown to the right. **B.** In a small number of cases, the selected seed were homozygous red, or green, fluorescent, which can be explained by formation of the F_2 individual from fertilisation of two independent crossover gametes. In these cases both crossover positions were retained for analysis. **C.** In these cases all genotypes were Col homozygous and are most likely explained by seed contamination, and so these samples were removed from analysis. **D.** Two samples showed an unexpected recombinant profile, consistent with three crossover events. This can be interpreted as one chromatid containing a single crossover producing a red-alone fluorescent phenotype. The other chromatid appears to have experienced a double crossover that resulted in a Col introgression into an otherwise Ler background.
