# Supplementary material for: Structural variation and DNA methylation shape the centromere-proximal meiotic crossover landscape in Arabidopsis
Source: Genome Biol. 2024 Jan 22;25:30. doi: 10.1186/s13059-024-03163-4 (PMC10804481; doi:10.1186/s13059-024-03163-4)
Supplement: Supplementary file 16 — Additional file 16: Figure S8. Chromatin and recombination states within CTL3.9 hotspots and coldspots. [file 13059_2024_3163_MOESM16_ESM.pdf]

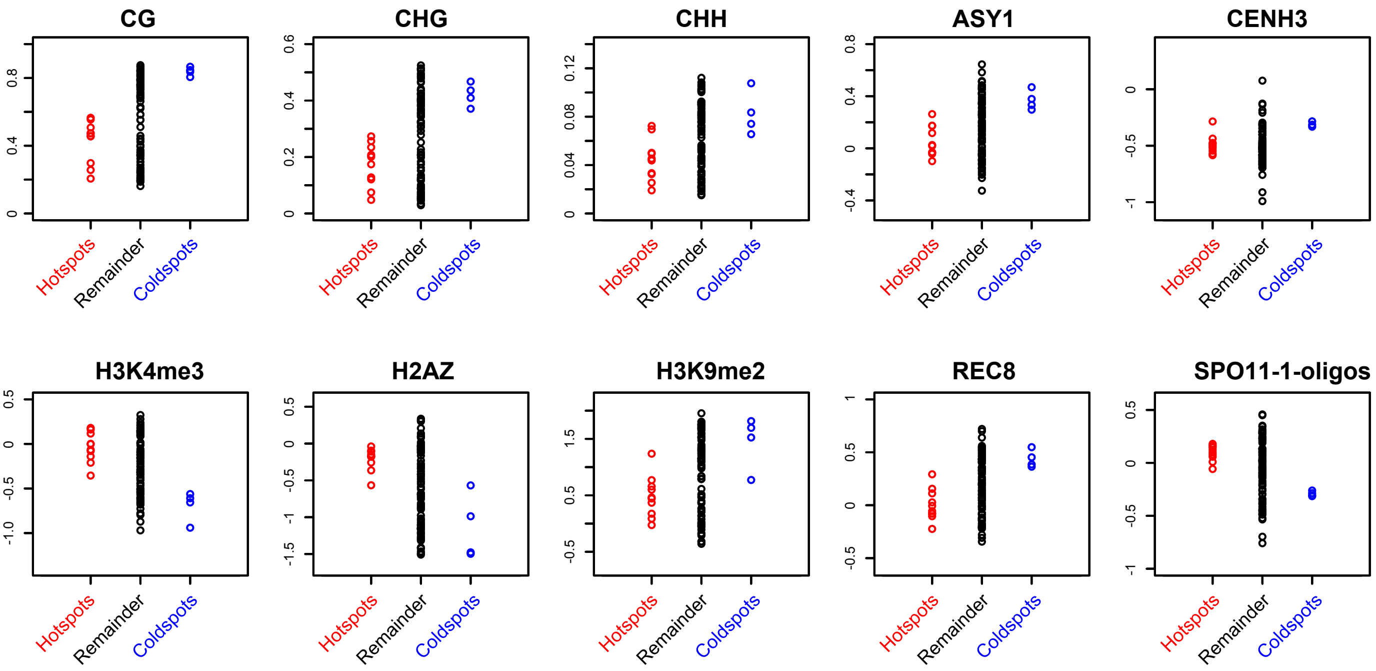
CG
0
0.4
0.8
Hotspots
Remainder
Coldspots
CHG
0
0.2
0.4
0.6
Hotspots
Remainder
Coldspots
CHH
0
0.04
0.08
0.12
Hotspots
Remainder
Coldspots
ASY1
-0.4
0
0.4
0.8
Hotspots
Remainder
Coldspots
CENH3
-1
-0.5
0
Hotspots
Remainder
Coldspots
H3K4me3
-1.0
-0.5
0
0.5
Hotspots
Remainder
Coldspots
H2AZ
-1.5
-1
-0.5
0
0.5
Hotspots
Remainder
Coldspots
H3K9me2
-0.5
0.5
1.5
Hotspots
Remainder
Coldspots
REC8
-0.5
0
0.5
1
Hotspots
Remainder
Coldspots
SPO11-1-oligos
-1
-0.5
0
0.5
Hotspots
Remainder
Coldspots

Additional file 8: Figure S8. Chromatin and recombination states within *CTL3.9* hotspots and coldspots. *CTL3.9* map intervals that had significantly higher (hotspots, red) or lower (coldspots, blue) crossover frequency were analysed for multiple chromatin and recombination datasets and compared with the remainder (black) of the map intervals. Intervals are compared for ONT-based DNA methylation levels in CG, CHG and CHH sequence contexts, ASY1, CENH3, H3K4me3, H2A.Z, H3K9me2, REC8 and SPO11-1-oligos. Information on chromatin datasets analysed is available in **Additional file 6: Table S2**.
