# Supplementary material for: Structural variation and DNA methylation shape the centromere-proximal meiotic crossover landscape in Arabidopsis
Source: Genome Biol. 2024 Jan 22;25:30. doi: 10.1186/s13059-024-03163-4 (PMC10804481; doi:10.1186/s13059-024-03163-4)
Supplement: Supplementary file 18 — Additional file 18: Figure S9. Structure of the HOTSPOT6 genetic interval in the Col and Ler genome assemblies. [file 13059_2024_3163_MOESM18_ESM.pdf]

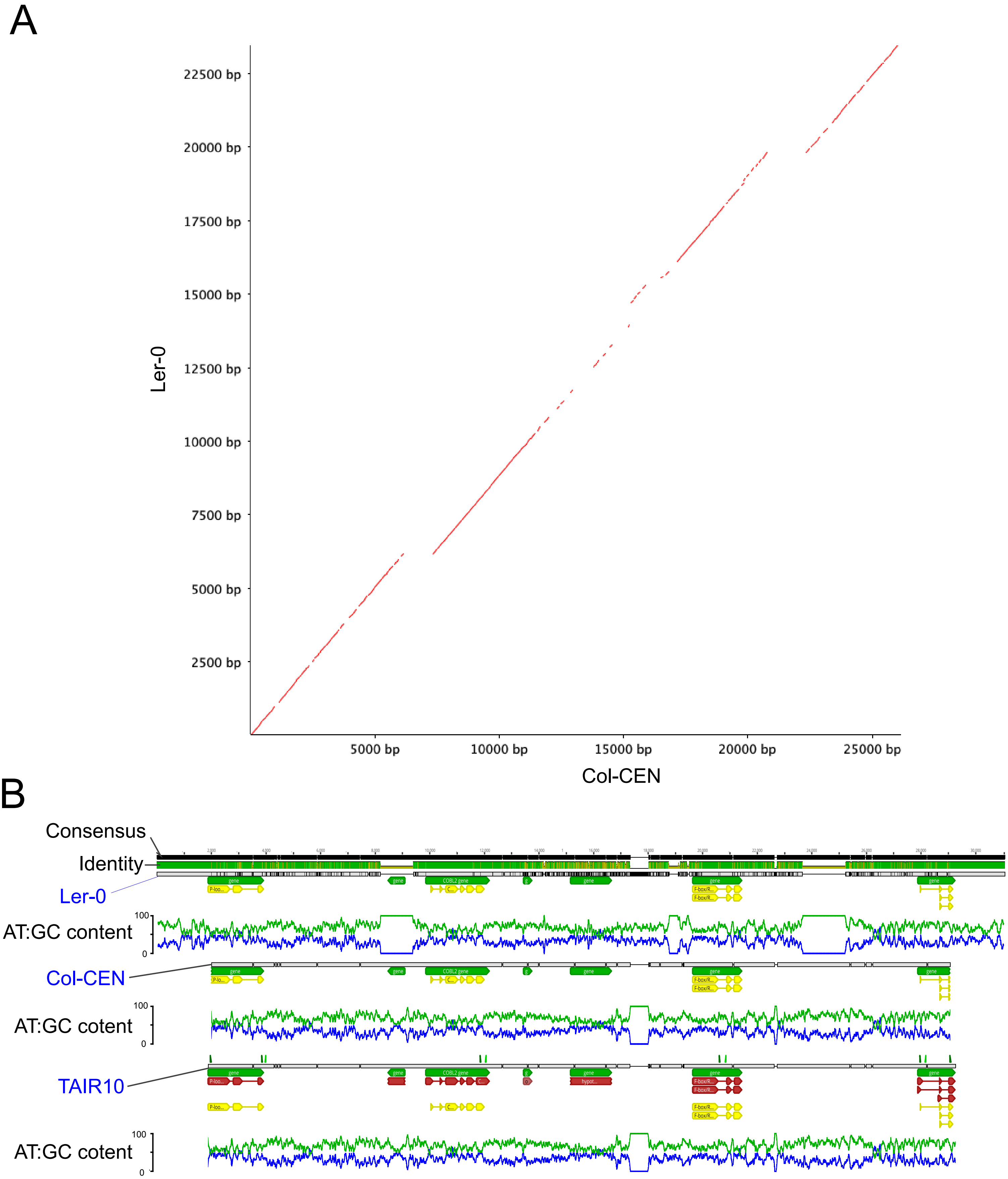

A
Ler-0
22500 bp
20000 bp
17500 bp
15000 bp
12500 bp
10000 bp
7500 bp
5000 bp
2500 bp
5000 bp
10000 bp
15000 bp
20000 bp
25000 bp
Col-CEN
B
Consensus
Identity
Ler-0
AT:GC content
100
0
Col-CEN
AT:GC cotent
TAIR10
AT:GC cotent

Additional file 18: Figure S9. Structure of the *HOTSPOT6* genetic interval in the Col and Ler genome assemblies. A. Sequence identity dotplot comparing the *HS6* interval in the Col-CEN and Ler-HiFi assemblies, using a 50 bp search window. **B.** Positions of the At3g29800, At3g29810, At3g29830 and At3g29970 genes in the Ler-HiFi, Col-CEN and TAIR10 genome assemblies, including plots of % AT (green) and GC (blue) base content over the region. Gene models are indicated as green arrows, with red and yellow annotations showing intron-exon structure.
