# Supplementary material for: Structural variation and DNA methylation shape the centromere-proximal meiotic crossover landscape in Arabidopsis
Source: Genome Biol. 2024 Jan 22;25:30. doi: 10.1186/s13059-024-03163-4 (PMC10804481; doi:10.1186/s13059-024-03163-4)
Supplement: Supplementary file 22 — Additional file 22:. Review history. [file 13059_2024_3163_MOESM22_ESM.docx]

Review History

**First round of review**

**Reviewer 1**

**Were you able to assess all statistics in the manuscript, including the appropriateness of statistical tests used?**

Yes, and I have assessed the statistics in my report.

**Comments to author:**

The manuscript presents a detailed analysis of recombination in the centromeric regions of Arabidopsis using improved CEN assemblies. The information and presentation are of high quality, shedding light on a previously inaccessible part of the genome. The authors utilize genome-wide recombination data for a genome wide analysis. Next, they focus on the centromere of chromosome 3 using closely flanking fluorescent marker lines. These lines enable the detection of COs (crossover events) in an 8Mb region encompassing CEN3. The CO reporter strain used in the study is well characterized (an important requirement), ruling out common chromosomal aberrations and determining the hybrid epigenetic state, particularly methylation. The authors observe opposing effects of MET1 and CMT3 methylases in regulating recombination and conclude that structural features and the epigenetic state suppress recombination.

The data presented strongly support the role of CG and CHG methylation in recombination. However, although structural differences are evident, their explicit effect on recombination is not tested. The authors missed an opportunity to carry out an experiment using the CTL3.9 line to measure COs between nearly identical copies of chromosome 3. This experiment could have tested the hypothesis that structural variation affects recombination. By introgressing the doubly marked Col CEN5 region into Ler and crossing it with the WT Col, an F1 hybrid could have been generated that would be homozygous Col for CEN3 and heterozygous for the fluorescent markers, while remaining hybrid for all other chromosomes. Such a comparison would have closely paralleled the full F1 hybrid described in the manuscript and potentially provided evidence for the central hypothesis. However, given the extensive work covered by the current results, demanding this experiment might be excessive. It is worth considering whether the authors are already conducting this experiment or if they could acknowledge that the hypothesis of structural effects on meiosis is supported by haplotypic characterization but not directly tested, thereby tempering their conclusions.

In Fig.1 and in other figs, the analysis is projected onto the ColCEN genome assembly, although recombination takes place in a the F1 hybrid Col/Ler. The choice makes some sense, given the importance of Col in genomic studies. However, one is left wondering about the epigenetic status of the hybrid, in which context recombination takes place. Arabidopsis accessions can have different epigenetic marks and their F1 can undergo additive and non-additive gene regulation. The relevance of ColCen choice vs Ler and the F1 should be discussed, at least in the Methods. What do genetic and recombination data look like when projected on the new T2T Ler genome? This question is addressed for chr3 and the CTL3.9 CO line in Fig. 4 and it seems comparable. So, while the authors leave out the hybrid context when looking at the whole genome, they are clearly aware of it and provide an excellent analysis of chr.3. In conclusion, please explain source of epigenetic marks when the data are projected onto ColCEN.

The number of CO used for each mutant analysis could be made clearer.

Fig. 1 C. Is the short arm of chr2 and chr3 treated as the regular arms? Is the same % identity scale used for StainedGlass plots on all five chromosomes? Please add bar or distribution plot.

Fig. 5. Is the gene annotation in A valid for Ler? Please clarify.

Fig. 6. I assume that the use of met1 +/- is due to the weakness and lethality in the homozygote. The additive effect of met1 wild-type alleles (i.e. haploinsufficiency) should be clarified for the reader.

Fig.S1-C. I am surprised that the SNPs/10kb are as low as indicated in the core centromeric regions given the obvious difference in CenHaps structure visible in Fig.4 and, even more clearly, in Fig S4. The manuscript text states that there is 1 SNP every few kb. In that case, I would expect a StainedGlass plot between Ler and Col and with identities threshold set as in Fig. S4 to yield a large red block. Perhaps I missed an obvious explanation. If not, please clarify.

Fig. S2, S3. Following the general manuscript convention, histone marks and other chromatin labels should be in red.

Fig. S5. A. The red and blue forward and reverse strand blocks in the CEN are confusing in the context of Fig.S4. What is this meant to convey?

It is not always clear what epigenetic data sets are derived from previous studies and what are determined in this work. The source is mentioned in some cases and not in others. References are cited, by the scope of the reference can be ambiguous. The source should be made explicit and access information should be provided in a supplementary table. If previously generated data are used (see, for example, Fig. S2, S7) were the original sequencing reads re-analyzed in the context of the ColCEN reference genome? This should be specified in Methods.

According to Methods, DNA methylation of Ler is derived from Nanopore sequencing. The method itself refers to both Ler and Col. Please change the heading to reflect this.

**Reviewer 2**

**Were you able to assess all statistics in the manuscript, including the appropriateness of statistical tests used?**

Yes, and I have assessed the statistics in my report.

**Comments to author:**

In this study, the authors define the boundaries of centromere-proximal crossover suppression in Arabidopsis thaliana and define two recombination zones: non-recombining zones (NRZs), with a complete absence of crossovers, and low-recombining zones (LRZs), within 1cM of the NRZ boundary. They go on to establish various features of these zones, including sequence composition, CENH3 enrichment, crossover rates, patterns of DNA methylation, heterochromatic histone modifications, GC content, gene, transposon, and SPO11-1-oligo density, etc. The authors observe structural polymorphisms between Col and Ler genomes and suggest that crossover exclusion in NRZs could be a direct result of these. The study fine-maps crossovers near CENTROMERE3 using fluorescent tagged lines, identifying hotspots and coldspots, and also that the recombination landscape calculated via this method correlates well with whole-genome sequencing techniques. Lastly, the study investigates genetic control of crossover rates within the CTL3.9 locus containing CENTROMERE3 and observes that a loss of CHG methylation leads to an increase in crossover rates, while a loss of CG methylation significantly reduces them. This would suggest that CHG and CG methylation control crossover rates in an antagonistic manner, with CHG contributing towards crossover suppress crossovers within the NRZ and CG contribution to crossover formation.

Overall, this was a well written and well organized paper that provides important genomic information about centromere-proximal regions of A. thaliana chromosomes, and investigates the genetic and epigenetic bases for crossover exclusion in these regions. The work is likely to be of interest to researchers in the fields of meiosis, recombination, epigenetics, and chromosome structure (including repetitive sequences, centromeres, etc.).

Minor points:

1. The authors' claim that structural polymorphisms between the Col/Ler hybrid are a potential direct reason for crossover suppression in NRZs seems to go against their supplemental data which shows that crossovers in the hybrid are significantly increased compared to the Col/Col inbred strains, where there are no structural polymorphisms. The FTL recombination data on the Col/Col should be moved into the main text and this point should be addressed.

2. It is entirely plausible that structure heterozygosity contributes to crossover suppression. However, in the case of inversions this will also lead to failure to recover crossover progeny. Can some of the observed reduction result from the fact that any crossovers that do take place between sequences that are inverted between Col and Ler will lead to dicentric and acentric products and will therefore not be seen among the progeny?

3. Although an interpretation is provided for met1 mutants showing increased centromere-proximal crossover suppression, how this relates to crossover patterns in the cmt3 mutant is not discussed.

4. Are there relevant mapping data from other organisms for comparison? Putting this work into a broader context will increase the appeal.

5. Many figures (3B, 3D, 3E, 3F, 4A, 4B, etc) are never referenced in the text.

**Authors Response**

**Point-by-point responses to the reviewers’ comments:**

*Please note that the numbering of several supplemental figures and tables has changed, due to addition of new data. In the responses, we refer to the updated figure and table numbers.*

**Reviewer #1:** 

**The manuscript presents a detailed analysis of recombination in the centromeric regions of Arabidopsis using improved CEN assemblies. The information and presentation are of high quality, shedding light on a previously inaccessible part of the genome. The authors utilize genome-wide recombination data for a genome wide analysis. Next, they focus on the centromere of chromosome 3 using closely flanking fluorescent marker lines. These lines enable the detection of COs (crossover events) in an 8Mb region encompassing CEN3. The CO reporter strain used in the study is well characterized (an important requirement), ruling out common chromosomal aberrations and determining the hybrid epigenetic state, particularly methylation. The authors observe opposing effects of MET1 and CMT3 methylases in regulating recombination and conclude that structural features and the epigenetic state suppress recombination.

The data presented strongly support the role of CG and CHG methylation in recombination. However, although structural differences are evident, their explicit effect on recombination is not tested. The authors missed an opportunity to carry out an experiment using the CTL3.9 line to measure COs between nearly identical copies of chromosome 3.**

*Reply: We note that in Figure 5C and Table S4 we present data analysing CTL3.9 crossover frequency in lines that are oth­erwise inbred Col/Col. The plants analysed had identical copies of all chromosomes, except for the CTL3.9 FTL T-DNA insertions, which were hemizygous. Therefore, a comparison of data from Figure 5C and Figure 5E provides a contrast of wild-type CTL3.9 recombination in a Col/Col inbred versus a Col/Ler hybrid background. Crossover frequency was significantly increased to 18.3 cM from 16.5 cM in an inbred Col/Col background. This is consistent with observations we previously reported in Blackwell et al. 2020 EMBO J, and Ziolkowski et al. 2015 eLife, where pericentromeric FTL intervals showed an increase in crossovers in hybrids* *compared to inbreds, in some, but not all, tested combinations.*

**This experiment could have tested the hypothesis that structural variation affects recombination. By introgressing the doubly marked Col CEN5 region into Ler and crossing it with the WT Col, an F1 hybrid could have been generated that would be homozygous Col for CEN3 and heterozygous for the fluorescent markers, while remaining hybrid for all other chromosomes. Such a comparison would have closely paralleled the full F1 hybrid described in the manuscript and potentially provided evidence for the central hypothesis. However, given the extensive work covered by the current results, demanding this experiment might be excessive. It is worth considering whether the authors are already conducting this experiment or if they could acknowledge that the hypothesis of structural effects on meiosis is supported by haplotypic characterization but not directly tested, thereby tempering their conclusions.**

*Reply: We agree that this is an interesting idea, but this would require multiple backcrosses and thus a substantial amount of time to fully investigate. In this study, we have completely assembled the two recombining Col and Ler haplotypes, where we observe a strong correlation between the presence of structural variation and suppression of crossover recombination within the centromere-proximal intervals. However, these structurally polymorphic intervals are also marked by specific chromatin states, including DNA methylation and CENH3 loading. We acknowledge that with the present data we cannot precisely delineate the relative contributions of structural variation and chromatin states to centromere-proximal recombination suppression, which is reflected in the paper title and the discussion where we propose that these factors jointly suppress crossovers.*

**In Fig.1 and in other figs, the analysis is projected onto the ColCEN genome assembly, although recombination takes place in a the F1 hybrid Col/Ler. The choice makes some sense, given the importance of Col in genomic studies. However, one is left wondering about the epigenetic status of the hybrid, in which context recombination takes place. Arabidopsis accessions can have different epigenetic marks and their F1 can undergo additive and non-additive gene regulation. The relevance of ColCen choice vs Ler and the F1 should be discussed, at least in the Methods.**

*Reply: While we have not measured chromatin states in Col/Ler F_1_ hybrids, instead, we have mapped DNA methylation via ONT sequencing, and CENH3 enrichment via ChIP-seq, against the Col-0 and Ler-0 homozygous parental strains. In both cases, data were mapped against complete assemblies for the matching accession. To make this clearer, we have added a sentence to the results at the end of page 13.*

**What do genetic and recombination data look like when projected on the new T2T Ler genome?**

*Reply: To address this point, we used the 14,397 crossover locations mapped against the Col-CEN genome and extracted the flanking 2 kb of sequences centred on each crossover midpoint. These sequences were aligned against the Ler-HiFi assembly using LASTZ, with only 4/14,397 that could not be aligned. For each crossover, we selected the alignment with the highest coverage value. Crossovers mapping to multiple loci with equally high coverage or identity values were removed, leaving a set of 13,958 high-confidence alignments. We next analysed the crossovers in 100 kb windows along the Ler-HiFi genome assembly, and calculated NRZ and LRZ coordinates, as before. The Ler NRZs extend over 16.3 Mb, compared to 17.8 Mb in Col, whereas the Ler LRZs are 13.9 Mb long, compared to 10.8 Mb in Col. We have added Col and Ler LRZ and NRZ coordinates to Table S1. We plotted the frequency of crossovers and SNPs against the Ler-HiFi assembly and have included this as a new Figure S2, which is reproduced below. Figure S2 can be compared to Figure S1, which shows an equivalent analysis against the Col-CEN genome. These results are now described in the main text and relevant information has been added to the Methods, describing this approach.*

*Reply: We have used in addition the Ler-HiFi crossover map to compare the Col and Ler LRZ-NRZ regions in a ‘butterfly’ format in Figure 4B; together with CENH3 ChIP-seq, DNA methylation, gene density and %GC content. These plots also include annotation of ATHILA retrotransposon insertions, and the location of regions of Col/Ler synteny and inversions determined by SyRI. This analysis highlights that all five centromeres in both Col and Ler are characterized by structurally polymorphic NRZs that contain specific chromatin states.*

**This question is addressed for chr3 and the CTL3.9 CO line in Fig. 4 and it seems comparable. So, while the authors leave out the hybrid context when looking at the whole genome, they are clearly aware of it and provide an excellent analysis of chr.3. In conclusion, please explain source of epigenetic marks when the data are projected onto ColCEN.**

*Reply: As explained in a later point, we provide a new Table S2 that lists the source of the chromatin data that were analysed against the Col-CEN and Ler-HiFi T2T assemblies. We have added a note to the relevant Figure legends referring to this table, and a similar note in the main text on page 8.*

**The number of CO used for each mutant analysis could be made clearer.** 

*Reply: For the SSLP data shown in Figure 5F (Table S11), 92 wild type, 90 HEI10 and 93 recq4a recq4b crossovers were analysed. These numbers are provided in the main text on page 16.*

*For the KASP mapping in Figure 6 (Tables S6-S7), we mapped 913 crossovers in wild type, 1,033 crossovers in cmt3 and 963 crossovers in met1/+. These numbers are provided at the end of the Introduction on page 5, and in the main text on pages 12 and 16.*

*For CTL3.9 analysis, the number of seeds analysed, and crossovers identified are provided in; Figure 5C (Table S4) (Col/Col wild type, cmt3, met1/+ and HEI10), Figure 5D (Table S5) (Col/Col wild type, atxr5 atxr6, ligase4, mom1 and smc4, and Figure 5E (Table S10) (Col/Ler wild type, cmt3, met1/+, HEI10 and recq4a recq4b).*

**Fig. 1 C. Is the short arm of chr2 and chr3 treated as the regular arms?**

*Reply: The analysis in Figure 1C in the originally submitted version included all ten chromosome arms. In Arabidopsis genomes, the left arms of chromosomes 2 and 4 are significantly shorter than the* *others. To test if their inclusion has a significant effect on the analysis presented in Figure 1C, we regenerated the plots subtracting the short arms of chromosomes 2 and 4. The regenerated plots (included below) did not differ substantially from the original version. Therefore, we would like to retain the original analysis that includes all ten chromosome arms in Figure 1C. We have modified the figure legend to clarify that the analysis includes all ten chromosome arms.*

**Is the same % identity scale used for StainedGlass plots on all five chromosomes? Please add bar or distribution plot.**

*Reply:**The plots represent independent % sequence identity scales. Histograms showing the % identity values and the corresponding colours have been added to Figure 1B to make this clearer.*

**Fig. 5. Is the gene annotation in A valid for Ler? Please clarify.** 

*Figure 5A provides an analysis of crossover distributions and gene annotation within the HOTSPOT6 (HS6) interval against the Col-CEN assembly.*

*Alongside, we show a sequence identity dot plot, using a 50 bp search window, comparing HS6 sequences from the Col-CEN (x axis) and Ler-HiFi (y axis) assemblies. The HS6 interval is largely syntenic between the assemblies, although there are some breaks in sequence similarity. This dot plot is included in Figure S9A.*

*We further used sequence alignment to manually annotate the positions of the genes in the HS6 interval (At3g29800, At3g29810, At3g29830 and At3g29970) in the Ler-HiFi assembly, in comparison with the Col-CEN and TAIR10 assemblies. This analysis, reproduced below, shows that gene order and arrangement within the HS6 interval is conserved between the Col-CEN and Ler-HiFi assemblies. We mention this in the main text on page 14. We have included the additional analyses in new Figure S9B.*

**Fig. 6. I assume that the use of met1 +/- is due to the weakness and lethality in the**

**homozygote. The additive effect of met1 wild-type alleles (i.e. haploinsufficiency) should be clarified for the reader.** 

*Reply: The reviewer is correct that met1 homozygotes show a strong developmental phenotype and reduced fertility that makes measurement of recombination using FTLs challenging. We have shown previously that met1/+ heterozygotes have reduced DNA methylation and changes to crossover frequency similar to those observed in met1 homozygotes (Yelina et al. 2015 Genes & Dev). This is proposed to reflect loss of maintenance methylation activity during haploid cell divisions in met1 gametophytes of met1/+ plants (Saze et al. 2003 Nat Genet). For these reasons we used met1/+ individuals to study CTL3.9 recombination. This is explained in the main text as follows; ‘CTL3.9 was crossed to generate met1-3/+ heterozygous plants, which have reduced crossovers comparable to met1-3 homozygotes yet have higher fertility* [*[45]*](https://paperpile.com/c/kaA6Hu/K4za)*.’.*
 **Fig.S1-C. I am surprised that the SNPs/10kb are as low as indicated in the core centromeric regions given the obvious difference in CenHaps structure visible in Fig.4 and, even more clearly, in Fig S4. The manuscript text states that there is 1 SNP every few kb. In that case, I would expect a StainedGlass plot between Ler and Col and with identities threshold set as in Fig. S4 to yield a large red block. Perhaps I missed an obvious explanation. If not, please clarify.**  

*Reply: In Figure S1, we plot the density of Col/Ler SNPs against the Col-CEN genome assembly. The SNPs were identified using SyRI (Goel et al., 2019). This method identifies SNPs through a pairwise whole-genome alignment, and syntenic regions with a unique one-to-one alignment are used to call biallelic SNPs. The StainedGlass plots in Figure S5 show a different type of analysis. For the StainedGlass analysis, adjacent 10 kb windows are subjected to all-vs-all alignment, and sequence identities of these alignments are displayed as a heat map coloured according to identity. Although the centromere regions are composed of the same family of CEN178 satellite repeats, their sequence, copy number and structural arrangement are very diverse between Col and Ler. These characteristics of core centromeric regions and their highly repetitive nature makes them unsuitable for defining syntenic/orthologous regions using SyRI. Therefore, it is not possible to define SNPs in the same manner as they are conventionally called in the chromosome arms. Hence, the lack of SNPs in the centromeres in Figure S1 reflects a technical difficulty in identifying polymorphisms in these repetitive regions. Instead, the sequence identity heat map comparisons in Figure S5 (and top and bottom of Figure 4A) provide a more informative comparison of similarities between Col and Ler centromeres for each chromosome. To make these points clear we have added a note to the legend of Figure S1.*

**Fig. S2, S3. Following the general manuscript convention, histone marks and other chromatin labels should be in red.** 

*Reply: Due to the large number of datasets analysed and plotted in this study, it is challenging to maintain a unified colour scheme throughout the manuscript. In Figures S3 and S4, our aim is to show CENH3 ChIP-seq enrichment (green) in relation to crossovers (cM/Mb, black), and multiple histone and other chromatin marks, which are shown in red. To further clarify the colour scheme used, we have made the y-axis text labels for the chromatin datasets match in red, whereas the CENH3 axis labels are in green, and the crossover labels are in black. The colour scheme is also clearly explained in the figure legends.*

**Fig. S5. A. The red and blue forward and reverse strand blocks in the CEN are confusing in the context of Fig.S4. What is this meant to convey?**

*Reply: The sequence identity dot plots shown in Figure S6 were generated by a different method to those in Figure S5. In Figure S6 we compared sequences using re-DOT-able (https://www.bioinformatics.babraham.ac.uk/projects/redotable/). This method uses a fixed window to test for sequence identity and adds shading in the dot plot where a match is found. The red and blue shading indicate forward and reverse strand similarity, respectively.*

*The StainedGlass method performs pairwise sequence alignments of a fixed window size and calculates % sequence identity, and then colors the cognate region of the dot plot according to the level of identity. The associated histogram provides a key for matching color and sequence identity levels. The advantage of StainedGlass is that it provides a more detailed image of varying levels of identity within the centromere repeat arrays. However, StainedGlass does not provide information on strand orientation. In the case of centromere 3, there is a large inversion within the satellite arrays, which is clear in the re-DOT-able heat map (Figure S6), but not in the StainedGlass plots (Fig. S5). The inversion within centromere 3 is also evident in Figure 3D.*

*To summarise, Figure S6A shows that the genome assembly of chromosome 3 is very similar between Col-CEN and the CTL3.9 line and does not contain structural rearrangements associated with the T-DNA insertions. In Figure S5, we provide a more detailed comparison of the centromeric satellite arrays between the Col-CEN and Ler-HiFi assemblies, for which purpose StainedGlass is a more appropriate tool. To cross-compare the methods Figure S6B analyses Col and Ler chromosome 3 using re-DOT-able, which can be compared with the StainedGlass comparisons in Figure S5 for this chromosome.*

**It is not always clear what epigenetic data sets are derived from previous studies and what are determined in this work. The source is mentioned in some cases and not in others. References are cited, by the scope of the reference can be ambiguous. The source should be made explicit and access information should be provided in a supplementary table. If previously generated data are used (see, for example, Fig. S2, S7) were the original sequencing reads re-analyzed in the context of the ColCEN reference genome? This should be specified in Methods.**

*Reply: Thank you for this suggestion. We have now added a new Table S2 that provides references for the datasets analysed in this study. These papers provide detailed information on data types and sources. All data sets were re-analysed in the context of the Col-CEN or the Ler-HiFi genome assembly*.

| Data type | Publication |
| --- | --- |
| Crossovers (Col/Ler) | [[9,33]](https://paperpile.com/c/kaA6Hu/Y0AZ+IyDZ) |
| CENH3 ChIP-seq (Col-0) | [[21]](https://paperpile.com/c/kaA6Hu/0i5G) |
| CENH3 ChIP-seq (Ler-0) | [[21]](https://paperpile.com/c/kaA6Hu/0i5G) |
| DNA methylation ONT (Col-0) | [[22]](https://paperpile.com/c/kaA6Hu/0i5G) |
| DNA methylation ONT (Ler-0) | [[21]](https://paperpile.com/c/kaA6Hu/0i5G) |
| DNA methylation (Col-0, *cmt3*, *met1*) | [[90,91]](https://paperpile.com/c/kaA6Hu/n6N2+xeBw) |
| RNA-seq (Col-0) | [[89]](https://paperpile.com/c/kaA6Hu/wWf7) |
| SPO11-1-oligos | [[40]](https://paperpile.com/c/kaA6Hu/ARGW) |
| ASY1 ChIP-seq | [[39]](https://paperpile.com/c/kaA6Hu/3RiH) |
| REC8 ChIP-seq | [[28]](https://paperpile.com/c/kaA6Hu/9WIx) |
| H3K9me2 ChIP-seq | [[28]](https://paperpile.com/c/kaA6Hu/9WIx) |
| H3K27me1 ChIP-seq | [[38]](https://paperpile.com/c/kaA6Hu/3PFV) |
| H2A.Z ChIP-seq | [[38]](https://paperpile.com/c/kaA6Hu/3PFV) |
| H2A.W6 ChIP-seq | [[38]](https://paperpile.com/c/kaA6Hu/3PFV) |
| H2A.W7 ChIP-seq | [[38]](https://paperpile.com/c/kaA6Hu/3PFV) |
| H3K27me3 ChIP-seq | [[38]](https://paperpile.com/c/kaA6Hu/3PFV) |

**According to Methods, DNA methylation of Ler is derived from Nanopore sequencing. The method itself refers to both Ler and Col. Please change the heading to reflect this.**  

*Reply: We have changed the heading to read ‘Profiling DNA methylation in using Oxford Nanopore sequencing’.*

**Reviewer #2:**

**In this study, the authors define the boundaries of centromere-proximal crossover suppression in Arabidopsis thaliana and define two recombination zones: non-recombining zones (NRZs), with a complete absence of crossovers, and low-recombining zones (LRZs), within 1cM of the NRZ boundary. They go on to establish various features of these zones, including sequence composition, CENH3 enrichment, crossover rates, patterns of DNA methylation, heterochromatic histone modifications, GC content, gene, transposon, and SPO11-1-oligo density, etc. The authors observe structural polymorphisms between Col and Ler genomes and suggest that crossover exclusion in NRZs could be a direct result of these.**

**The study fine-maps crossovers near CENTROMERE3 using fluorescent tagged lines, identifying hotspots and coldspots, and also that the recombination landscape calculated via this method correlates well with whole-genome sequencing techniques. Lastly, the study investigates genetic control of crossover rates within the CTL3.9 locus containing CENTROMERE3 and observes that a loss of CHG methylation leads to an increase in crossover rates, while a loss of CG methylation significantly reduces them. This would suggest that CHG and CG methylation control crossover rates in an antagonistic manner, with CHG contributing towards crossover suppress crossovers within the NRZ and CG contribution to crossover formation. 

Overall, this was a well written and well organized paper that provides important genomic information about centromere-proximal regions of *A. thaliana* chromosomes, and investigates the genetic and epigenetic bases for crossover exclusion in these regions. The work is likely to be of interest to researchers in the fields of meiosis, recombination, epigenetics, and chromosome structure (including repetitive sequences, centromeres, etc.).

Minor points:

1. The authors' claim that structural polymorphisms between the Col/Ler hybrid are a potential direct reason for crossover suppression in NRZs seems to go against their supplemental data which shows that crossovers in the hybrid are significantly increased compared to the Col/Col inbred strains, where there are no structural polymorphisms. The FTL recombination data on the Col/Col should be moved into the main text and this point should be addressed.**

*Reply: Data on CTL3.9 FTL crossover frequency in an otherwise Col/Col inbred background are shown in Figure 5C for wild type, cmt3, met1/+ and HEI10 genotypes. This can be compared to the CTL3.9 Col/Ler wild type hybrid in Figure 5E. As noted by the reviewer, CTL3.9 shows a significant increase in crossover frequency from 16.5 cM in an inbred background to 18.3 cM in Col/Ler hybrids.* *In the text, we refer to observations previously made in Blackwell et al 2020 EMBO J, and Ziolkowski et al 2015 eLife, where we reported that pericentromeric FTL intervals show an increase in crossovers in some, but not all, combinations of F_1_ hybrids.*

**2. It is entirely plausible that structure heterozygosity contributes to crossover suppression. However, in the case of inversions this will also lead to failure to recover crossover progeny. Can some of the observed reduction result from the fact that any crossovers that do take place between sequences that are inverted between Col and Ler will lead to dicentric and acentric products and will therefore not be seen among the progeny?** 

*Reply: We acknowledge that there are inversions between the Col and Ler genome, including within, or in proximity to CEN3, CEN4 and CEN5, which are highlighted as pink boxes in Figure 1A and Figure 4B. We agree with the reviewer that crossovers occurring in these inversions could produce acentric and dicentric products that would not be represented in the progeny. However, CEN1 and CEN2, and large regions of the NRZs on the other chromosomes lack inversions. Therefore, we do not think that inversions explain the suppression of centromeric crossovers seen in the majority of the NRZ regions. We acknowledge that any crossovers that formed within the inversions would likely not be visible in the progeny. We propose that suppression of centromere-proximal crossovers occurs jointly because of both structural polymorphisms, including inversions, and chromatin states including high DNA methylation and CENH3 occupancy.*

**3. Although an interpretation is provided for *met1* mutants showing increased centromere-proximal crossover suppression, how this relates to crossover patterns in the cmt3 mutant is not discussed.**

*Reply: The met1 and cmt3 mutants are similar, as both show increased levels of meiotic DSBs close to the centromere, as measured by SPO11-1-oligos (Choi et al 2018 Genome Research.; Underwood et al 2018 Genome Research). Yet, met1 and cmt3 show opposite effects on centromere-proximal crossovers, which are reduced and increased, respectively. The met1 and cmt3 mutants are distinguished in at least two ways at the chromatin level; (i) H3K9me2 is largely intact in met1 (Deleris et al 2012 PLoS Genetics), but is reduced in cmt3 (Stroud et al 2014 NSMB), and (ii) in cmt3, CG and CHH methylation are intact, but CHG is very reduced, whereas in met1 CG is globally reduced and CHG/CHH methylation are also reduced within the CEN178 arrays (Stroud et al 2013 Cell; Naish et al 2021 Science). We propose that due to these distinct chromatin states, in the centromere-proximal regions of cmt3 more meiotic DSBs are capable of maturing into crossovers than in wild type. In met1, meiotic DSBs are also increased in proximity to the centromere, but we propose that the distinct chromatin state causes a greater zone of crossover inhibition around the centromere, meaning overall crossover levels are reduced, despite an increase in the initiating DSBs. We have added further material to the Discussion to communicate these aspects.*

**4. Are there relevant mapping data from other organisms for comparison? Putting this work into a broader context will increase the appeal.**

*Reply: To further place our work in context, we now include additional discussion of recent work on centromere-proximal recombination in Drosophila (Hartmann et al. 2019 Genetics):*

*“In Drosophila melanogaster, crossovers are strongly suppressed in the centromere-core, in addition to flanking pericentromeric heterochromatin* [*[71]*](https://paperpile.com/c/L3ANuH/OoYe)*. Interestingly, in Drosophila Blm mutants, the centromere-core remains crossover-suppressed, but the flanking low-recombining zones increase recombination, despite normal levels of heterochromatin* [*[71]*](https://paperpile.com/c/L3ANuH/OoYe)*. As the Blm mutant disrupts multiple aspects of crossover patterning in Drosophila* [*[72]*](https://paperpile.com/c/L3ANuH/M78O)*, this is consistent with centromere-proximal suppression of recombination involving long-range communication”.*

*Our discussion already included comparison of our findings to other eukaryotes including budding yeast, fission yeast and human. For example,*

*“As the centromeric and pericentromeric regions are enriched for REC8-cohesin* [*[28]*](https://paperpile.com/c/kaA6Hu/9WIx)*, by analogy with budding yeast this may promote meiotic DSB repair using a sister chromatid, contributing to crossover suppression* [*[8,68]*](https://paperpile.com/c/kaA6Hu/g8sr+xXbL)*.”*

and,

*“In comparison, human centromeres are composed of similar megabase-scale α-satellite arrays, although narrower (~100 kb) regions of CENP-A enrichment are observed, which are DNA hypomethylated in the CG sequence context* [*[23,25]*](https://paperpile.com/c/kaA6Hu/THzK+9Una)*. As human centromeres are also suppressed for meiotic crossovers* [*[10]*](https://paperpile.com/c/kaA6Hu/DJSC)*, species-specific chromatin and DNA sequence organisation may play varying roles in shaping centromere-proximal recombination landscapes.”*

**5. Many figures (3B, 3D, 3E, 3F, 4A, 4B, etc) are never referenced in the text.**

*Reply: We have added references to these figures at appropriate places in the text: Fig 1C, Fig 3B*

*Fig 3C, Fig 3D Fig 3E, Fig 3F Fig 3G Fig 4A and Fig 4B.*

**Second round of review**

**Reviewer 2**

The revisions and responses have addressed all of my comments, and the authors seem to have addressed the comments of the other reviewer adequately. There are of course many experiments I would like to see done, but this work already tells a nice story with the substantial data and analysis within.
